# Small-molecule PTPN2 Inhibitors Sensitize Resistant Melanoma to Anti-PD-1 Immunotherapy

**DOI:** 10.1158/2767-9764.CRC-21-0186

**Published:** 2023-01-24

**Authors:** Zhouting Zhu, Rachel Tang, Sarah Huff, Indrasena Reddy Kummetha, Lingling Wang, Na Li, Tariq M. Rana

**Affiliations:** 1 Division of Genetics, Department of Pediatrics, Program in Immunology, Institute for Genomic Medicine, University of California San Diego, La Jolla, California.; 2 San Diego Center for Precision Immunotherapy, Moores Cancer Center, University of California San Diego, La Jolla, California.

## Abstract

Although immune checkpoint inhibitors targeting T-cell immunoregulatory proteins have revolutionized cancer treatment, they are effective only in a limited number of patients, and new strategies are needed to enhance tumor responses to immunotherapies. Deletion of protein tyrosine phosphatase non-receptor type 2 (Ptpn2), a regulator of growth factor and cytokine signaling pathways, has been shown to sensitize murine B16F10 melanoma cells to IFNγ and anti-PD-1 immunotherapy. Here, we investigated the potential therapeutic utility of small-molecule PTPN2 inhibitors. Ten inhibitors were synthesized on the basis of *in silico* modeling and structure-based design and functionally tested *in vitro* and *in vivo*. We show that the inhibitors had little effect on B16F10 cells alone, but effectively sensitized the tumor cells to IFNγ treatment *in vitro* and to anti-PD-1 therapy *in vivo.* Under both conditions, Ptpn2 inhibitor cotreatment suppressed B16F10 cell growth and enhanced Stat1 phosphorylation and expression of IFNγ response genes. *In vivo,* PTPN2 inhibitor cotreatment significantly reduced melanoma and colorectal tumor growth and enhanced mouse survival compared with anti-PD-1 treatment alone, and this was accompanied by increased tumor infiltration by granzyme B^+^ CD8^+^ T cells. Similar results were obtained with representative murine and human colon cancer and lung cancer cell lines. Collectively, these results demonstrate that small-molecule inhibitors of PTPN2 may have clinical utility as sensitizing agents for immunotherapy-resistant cancers.

**Significance:**

To enhance the effectiveness of immunotherapies in resistant or nonresponsive cancers, it is important to develop inhibitors of enzymes that negatively influence the outcome of treatments. We have designed and evaluated small-molecule inhibitors of PTPN2 demonstrating that these compounds may have clinical utility as sensitizing agents for immunotherapy-resistant cancers.

## Introduction

Many tumors escape destruction by the immune system by engaging inhibitory receptors expressed by T cells, such as CTL antigen 4 (CTLA-4) and programmed cell death protein 1 (PD-1; refs. 1, 2). Physiologically, engagement of these T-cell receptors by their ligands CD80/CD86 (CTLA-4) and PD-L1/2 (PD-1), respectively, expressed on antigen-presenting cells and other immune cell types serves to regulate T-cell activation and ensure self-tolerance. When expressed on tumor cells, however, the ligands effectively inhibit T-cell signaling and promote tolerance and exhaustion, enabling immune evasion and promoting tumor cell survival (1, 3). The development of antibodies and fusion proteins targeting the PD-1, PD-L1, and CTLA-4, collectively termed immune checkpoint inhibitors (ICI), has revolutionized cancer therapy, and their use as single-agent or combination therapies has significantly improved the outcomes for patients with a wide range of cancers (4, 5). However, for multiple reasons, ICIs are effective only in a subset of patients and those who do respond often develop resistance and experience relapse (6). Thus, there is a clear need for strategies that sensitize resistant tumors to ICIs to overcome the current limitations of this breakthrough class of therapies.

Engagement of PD-1 and CTLA-4 on T cells induces signaling events that counteract costimulatory receptor-induced kinase signaling and inhibit T-cell activation (1, 3). Protein tyrosine phosphatase non-receptor type 2 (PTPN2, also known as TCPTP) is a negative regulator of T-cell antigen receptor signaling and acts by dephosphorylating and inactivating Src family kinases (7, 8). In addition, PTPN2 inhibits signaling by a number of cytokines that play essential roles in T-cell differentiation, function, and homeostasis via dephosphorylation and inactivation of JAK1 and JAK3 and their target substrates STAT1, STAT3, and STAT5 (9–12). Ptpn2-mediated dephosphorylation of Stat1 and Jak1 negatively regulates signaling induced by IFNγ, a potent inhibitor of tumor growth via stimulation of the antitumor immune response (13–16). Previous work demonstrated that Ptpn2 silencing in B16F10 melanoma cells sensitized them to anti-PD-1 therapy *in vivo* by increasing IFNγ-induced antigen presentation and T-cell activation, culminating in inhibition of tumor growth (17). These results indicated that inhibition of Ptpn2 may be a feasible target for sensitizing tumors to immunotherapy *via* amplification of the IFNγ response.

Small-molecule drugs have many advantages as therapeutic agents over proteins which are costly and complex to produce and require special handling and parenteral administration. Here, we designed several small-molecule PTPN2 inhibitors using *in silico* modeling and structure-based design and selected 10 inhibitors for functional analysis. We examined the effect of the PTPN2 inhibitors on the IFNγ signaling response of mouse melanoma and colon cancer cell lines *in vitro* by comparing the effectiveness of the PTPN2 inhibitors with results of CRISPR/Cas9-mediated *Ptpn2* deletion. We also determined the lead inhibitors’ efficacy and mechanism of action *in vivo* using two syngeneic mouse models for melanoma and colorectal cancer. Overall, our results suggest that small-molecule inhibitors of PTPN2 may have utility as sensitizing agents to circumvent tumor resistance to ICIs.

## Materials and Methods

Detailed *in silico* modeling and structure-based design of Ptpn2 inhibitors, synthetic procedures, and compound characterizations are presented in the Supplementary Data.

### Cell Culture

B16F10 (CRL‐6475; murine melanoma), CT26 (CRL‐2638; murine colon carcinoma), HT29 (HTB-38, human colorectal adenocarcinoma), A549 (CCL-185, human lung adenocarcinoma), and CCD 841 CoN (CRL-1790, normal human colon tissue) were purchased from ATCC. MC38 (murine colon carcinoma) was purchased from Kerafast. Primary human T cells were isolated from peripheral blood mononuclear cells obtained from San Diego Blood Bank. Epithelial cells were grown and maintained in DMEM (Gibco) or RPMI (Gibco) supplemented with 10% of heat-inactivated FBS (Gibco), 2 mmol/L of l-glutamine, and 100 U/mL penicillin streptomycin (Gibco). T cells were grown in ImmunoCult-XF T Cell Expansion Medium (STEMCELL) supplemented with 300 U/mL recombinant human IL2 (STEMCELL) and 100 U/mL penicillin streptomycin. All cell lines were cultured in 37°C incubator with humidified atmosphere of 5% carbon dioxide. Trypsin-Ethylenediaminetetraacetic acid (EDTA) (1X) was used to detach adherent cells from culturing plate surface.

### Generation of CRISPR-knockout Tumor Cell Lines

#### Generation of the Lentivirus Vector

Lentivirus vectors were generated according to the protocol described in Shalem and colleagues (18). Oligonucleotides were synthesized with the target guide sequence (Supplementary Table S2), digested, annealed, and cloned into the lentiCRISPR v2 vector. Plasmids were transformed into Stbl3 bacteria and cultured on agar plates. The cultures were then grown in LB broth inoculated with ampicillin (100 μg/mL) for 12 hours. Plasmids were then purified from bacteria using Qiaprep Spin Miniprep Kit.

#### Transfection

Lentiviral production was performed in a 6-well culture plate using HEK293T cells. Transfection reagents were separated into Tube A and Tube B where Tube A contained Lipofectamine 2000 Reagent (Invitrogen) and Opti-MEM (Thermo Fisher Scientific). Tube B had both packaging plasmids (PsPAX and PMD2.G) and lentivector with ratio of 3:1:4, respectively. Both mixtures were vortex until homogenous. Immediately, both tubes were combined and incubated under room temperature for 15–20 minutes. Then, the transfection complex was transferred into HEK293T cells to incubate for 48 hours before collecting the virus.

#### Transduction

Cells were seeded into a 6-well culturing plate where the day of transduction achieves about 70% confluency. In each well, lentivirus and Polybrene (Millipore-Sigma) were added. Spin-transfection transduction was performed for 1.5 hours under 2,000 rpm. The following day (after 24 hours incubation), the media in each well was replaced with fresh media containing 1 to 10 μg/mL Puromycin (Alfa Aesar) for positive selection.

### *In Vitro* Cytokine Stimulation and Inhibitor Treatment

Cells were seeded a day before in a 12-well plate stimulation with cytokines where 50% confluency was achieved the following day. The two cytokines being used were Recombinant Mouse IFNγ (Animal-Free) Protein (BioLegend) and TNFα (Carrier-Free) Protein (BioLegend). Cells were seeded on day 0 in a 12-well culture to achieve 50% confluency by day 1. The culture medium was replaced on day 1 with the medium containing 30 μmol/L of indicated inhibitor or same volume of DMSO, with or without IFNγ. The cells were further incubated for 72 hours and analyzed on day 4. For IFNγ + TNFα stimulation, same methods, except for the treatment with or without combination of IFNγ + TNFα, were applied.

### RNA Extraction and qRT-PCR

Total RNA was isolated from cells using TRIzol Reagent (Invitrogen) and subjected (1,000 ng RNA) to reverse transcription with iScript Reverse Transcription Supermix (Bio-Rad) for cDNA generation following manufacturer's protocol. qRT-PCR was performed via iTaq Universal SYBR Green Supermix (Bio-Rad) using Roche's Light Cycler 480 II. Normalization and fold changes for each gene's expression were quantified using the comparative *C*_t_ method with GAPDH as an internal control. Primers are listed in Supplementary Table S3.

### Cytokine Treatment Proliferation Assay and MTS Tetrazolium Assay

Cell numbers after cytokines treatment were calculated using hemocytometer. Dead and live cells were differentiated with Trypan Blue 0.4% (Lonza). For MTS assay, cells were seeded in a 96-well plate one day before drug stimulation. The cellular viability was determined using CellTiter96 Aqueous One Solution Reagent (Promega), measuring the absorbance of tetrazolium compound MTS [3-(4,5-dimethylthiazol-2-yl)-5-(3-carboxymethoxyphenyl)-2-(4-sulfophenyl)-2H-tetrazolium, inner salt] at 490 nm. 20 μL of MTS reagent was added into each well for 1–4 hours incubation at 37°C. Absorbance was measured using a Synergy 2 BioTek plate reader.

### Protein Extraction and Western Blotting

Cells were lysed in lysis buffer (60 mmol/L Tris HCL, 2% SDS, and 10% glycerol) containing Benzoate Nuclease (Sigma-Aldrich), dithiothreitol (DTT), phenylmethylsulfonylfluoride, and Protease Inhibitor Cocktail 3 Mammalian (RPI). Lysate were incubated on ice for 15 minutes and then centrifuged at 13,000 rpm for 20 minutes at 4°C. Protein supernatant concentration was quantified using DC Protein Assay (Bio-Rad) with Synergy 2 BioTek plate reader measuring at 750 nm. A total of 50–80 μg of proteins with LDS loading buffer (Thermo Fisher Scientific) were denatured at 95°C for 8 minutes before loading into NuPAGE Novex 4%–12% Bis-Tris Gel 1.5 mm (Invitrogen). Ladder used to estimate protein size was SeeBlue Plus2 Prestained Protein Standard (Thermo Fisher Scientific). After the blot transfer (Trans-blot Turbo, Bio-Rad), polyvinylidene difluoride membranes (Bio-Rad) were cut and incubated overnight with 1% BSA and designated antibodies, followed by membrane washes with phosphate-buffered saline with Tween 20 detergent (PBST) (Bio-Rad), horseradish peroxidase (HRP)-linked second antibody incubation, and film (Thermo Fisher Scientific) exposure. Antibodies: Phospho-Stat1 (Tyr701; D4A7) Rabbit mAb Antibody (Cell Signaling Technology, 1:1,300), Stat1 Rabbit mAb Antibody (Cell Signaling Technology, 1:1,300), Anti-rabbit IgG, HRP-linked Antibody (Cell Signaling Technology, 1:1,000), and GAPDH (14C10) Rabbit mAb (HRP Conjugate) Antibody (Cell Signaling Technology, 1:1,000).

### Mice and Treatments

All procedures were approved by the University of California, San Diego Institutional Animal Care and Use Committee.

For B16F10 melanoma, 7 to 9 weeks old wildtype female C57BL/6J mice were obtained from the Jackson laboratory. Mice were age matched to be 7–12 weeks old at the time of tumor inoculation. A total of 0.5 × 10^6^ B16F10 melanoma cells were resuspended in PBS (Gibco) and subcutaneously injected to the right flank of mice on day 0. On days 1 and 4, mice were vaccinated with irradiated (100 Gy) GVAX cells on the left flank to elicit an antitumor immune response. After 5 days, mice were randomized into inhibitor or DMSO control treatment groups when the tumor volumes reached 150 mm^3^. On days 6, 9, and 12, all mice were intraperitoneally injected with 200 μg PD-1 antibody (10 mg/kg). Ptpn2 inhibitor (50 mg/kg diluted in DMSO, 10 μL per mouse) or DMSO (10 μL per mouse) was intratumorally injected to the two groups on day 10, 12, and 14. Tumors were measured every 2 days from day 7 until the time of death or day 18.

For colon cancer CT26, 6 to 9 weeks old wildtype female Balb/c mice were obtained from the Jackson laboratory. Mice were age matched to be 7–12 weeks old at the time of tumor inoculation. A total of 2 × 10^6^ CT26 cells were resuspended in PBS and Matrigel (Thermo Fisher Scientific) at the ratio of 1:1, and then subcutaneously injected to the right flank of mice on day 0. After 9 days, mice were randomized into inhibitor or DMSO control treatment groups when the tumor volumes reached 80 mm^3^. Starting from day 11, all mice were intraperitoneally injected with PD-1 antibody (200 μg per mouse) every 4 days, followed by Ptpn2 inhibitor (50 mg/kg diluted in DMSO, 10 μL per mouse) or DMSO (10 μL per mouse) intratumoral injection. Tumors were measured starting from day 11 along with PD-1 antibody treatment until the time of death.

Date of death was defined as the day the tumor reached 2.0 cm in the longest dimension. Tumor volume = (length × width^2^)/2. Mice were euthanized with CO_2_ inhalation on the day of euthanasia. On the day of harvest, tumors were collected blindly by other personnel without knowing the treatment to minimize the subjective biases. For Western blotting, qRT-PCR and flow cytometry analysis, broken, bleeding, extreme small samples (<60 mm^3^) were excluded. Unless recording survival curve, all the tumors were preserved for further analyses.

### Flow Cytometry Analysis of Tumor-infiltrating Lymphocytes

B16F10 melanoma tumors were dissected on either the day when the tumor reached 2.0 cm length or day 18. The tumor tissues were weighed, mechanically diced, incubated with collagenase P (2 mg/mL, Sigma-Aldrich) and DNase I (50 μg/mL, Sigma-Aldrich) for 15 minutes and then pipetted into a single-cell suspension. Cells were filtered through a 70 μmol/L filter (Corning). Anti-mouse CD16/32 antibody (BioLegend) was used to block all samples. Dead cells were excluded by Zombie Aqua (BioLegend). All surface and intracellular markers were stained under per manufacturer's instruction. Antibodies used for flow cytometry were purchased from BioLegend and included CD45 (clone 30-F11), CD8 (clone 53-6.7), CD4 (clone RM4-5), CD3ε (clone 145-2C11), granzyme B (clone 25-8898-82). Single-color compensation controls and fluorescence-minus-one thresholds were used on RUO green to set gate margins.

### Statistical Analysis

GraphPad Prism v.8.0a program was used to perform all statistical test and graphing of data. Data were presented as mean ± SEM (standard error) or mean ± SD as indicated. *P* values were calculated using either Student *t* test or two-way ANOVA. *P* < 0.05 was considered statistically significant.

### Study Approval

This study was approved by the University of California San Diego (UCSD) Institutional Review Board. All procedures were approved by the UCSD Institutional Animal Care and Use Committee.

### Data and Materials Availability

The data and materials generated in this study are available upon request from the corresponding author.

## Results

### *In Silico* Modeling and Structure-based Design of PTPN2 Inhibitors

**FIGURE 1:**
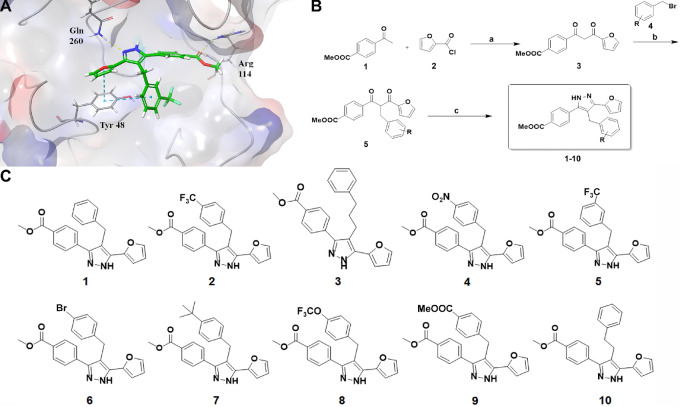
Design and synthesis of PTPN2 inhibitors. **A,** Molecular docking model of inhibitor 5 bound to the catalytic PTP domain of PTPN2 (PDB ID: 1L8K; ref. 26). Inhibitor 5 is predicted to form hydrogen bonds with Arg 114 and Gln 260. Tyr 48 is expected to form π-π stacking interactions with two aromatic rings of inhibitor 5. **B,** Synthetic scheme for PTPN2 inhibitors. **A,** LiHMDS, THF, 0°C to room temperature, 12 hours. **B,** K_2_CO_3_, NaI, acetone, reflux, 12 hours. **C,** N_2_H_4_, EtOH, 80°C, 2 hours. **C,** Structures of Ptpn2 inhibitors 1–10.

PTPN2 is a member of the classical, non-receptor protein tyrosine phosphatase (PTP) superfamily and catalysis is mediated by a highly conserved PTP domain containing the signature HCX_5_R loop motif (19, 20). Despite high sequence conservation across the PTP superfamily, selective small-molecule inhibitors of phosphatase activity have successfully been developed for the homologous proteins PTP1B and SHP2/PTPN11 by exploiting small sequence variations in the periphery of the catalytic domain (21–24). We based our design on the inhibitor PHPS1, which shows selective inhibition of SHP2 compared with DUSP18, DUSP23, DUSP26, PTP1B, and SHP1 phosphatases (25). Using the Schrödinger software suite (26) and Glide XP (27–29), we performed docking using the X-ray crystal structure of the PTPN2 catalytic domain (PDB ID: 1L8K; Fig. 1A), and Qikprop was used to calculate the physicochemical properties (including clogP, PSA, SASA, and expected permeability in Caco-2 and MDCK models) of the compounds prior to docking (Supplementary Table S1). Compounds expected to be cell permeable were prioritized for synthesis. Docking poses were evaluated for interactions with both the conserved HCX_5_R motif and with residues at the periphery of the catalytic site, including Tyr 48 and Gln 260 (Fig. 1A). The top 10 inhibitors (referred to as PTP 1 through PTP 10) that satisfied the selection criteria were selected for synthesis according to Scheme 1 (Fig. 1B and C).

### CRISPR/Cas9-mediated *Ptpn2* Knockout Sensitizes Melanoma Cells to IFNγ Treatment

**FIGURE 2:**
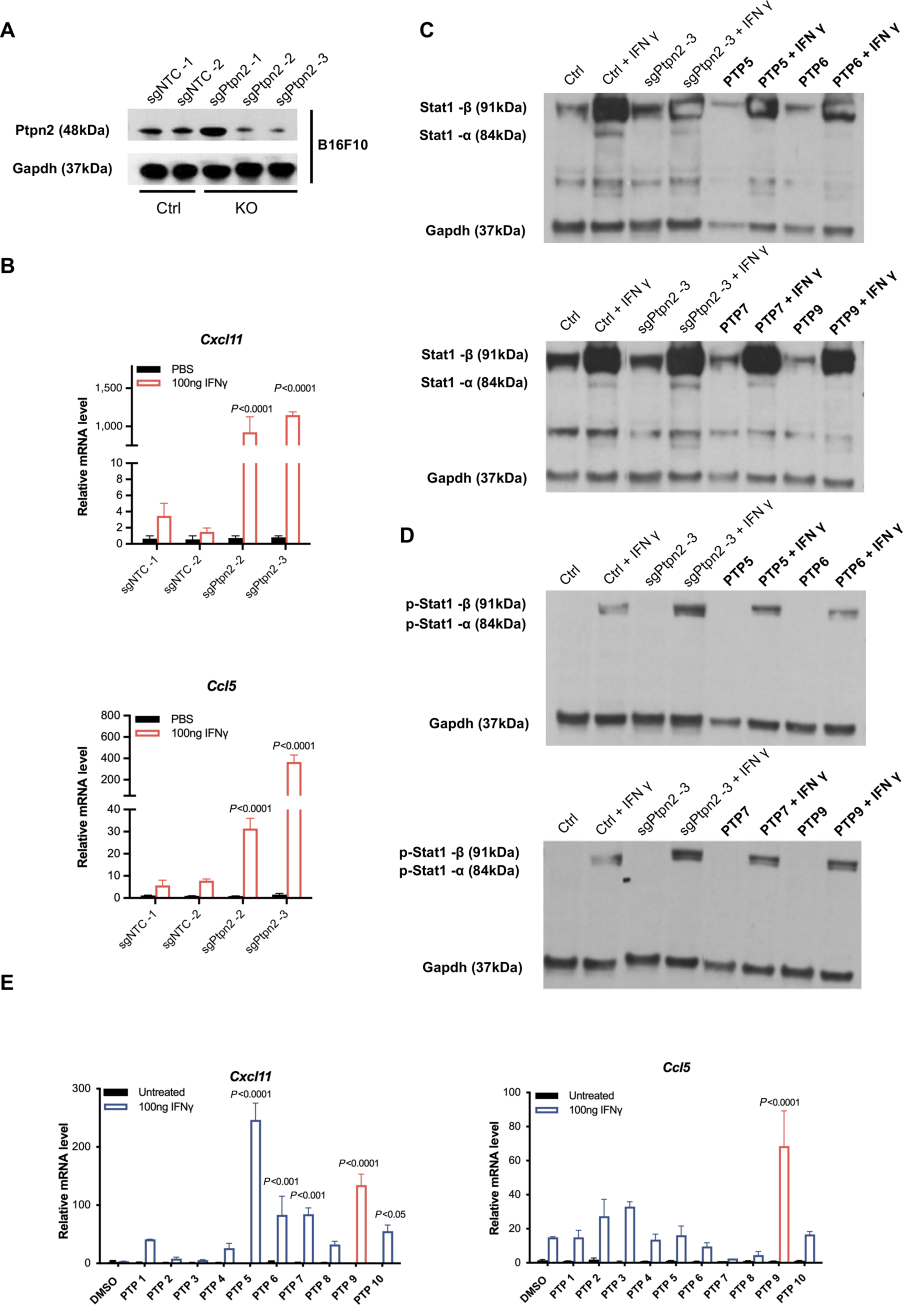
PTPN2 inhibitors sensitize B16F10 cells to IFNγ treatment. **A,** Western blot analysis of Ptpn2 expression in control (sgNTC) or *Ptpn2* knockout (sgPtpn2) B16F10 melanoma cells. **B,** qRT-PCR analysis of Cxcl11 and Ccl5 mRNA in control or *Ptpn2* knockout B16F10 cells incubated for 24 hours with PBS or 100 ng/mL IFNγ. *P* values compare *Ptpn2* knockout with the control both under IFNγ treatment by Student *t* test. Data are the mean ± SEM of *n* = 4. **C,** Western blot analysis of Stat1 in B16F10 cells treated as indicated. **D,** Western blot analysis of phosphorylated Stat1 in B16F10 cells treated as indicated. **E,** qRT-PCR analysis of Cxcl11 and Ccl5 mRNA in B16F10 cells incubated for 24 hours with PBS or 100 ng/mL IFNγ, together with DMSO or 30 μmol/L of the indicated Ptpn2 inhibitors. *P* values compare inhibitors to the DMSO control under IFNγ treatment by Student *t* test. Data are the mean ± SEM of *n* = 2.

Previous reports showed that PTPN2 negatively regulates IFNγ signaling by inhibiting dephosphorylation of Jak1 and Stat1 (13–16). We theorized that *Ptpn2* inhibition might sensitize tumor cells to ICI treatment by increasing IFNγ signaling, as reported previously (17). To this end, we generated control and *Ptpn2* knockout B16F10 murine melanoma cell line by CRISPR/Cas9 editing (18, 30, 31). Stable cell lines were produced, the efficiency of *Ptpn2* knockout was confirmed by Western blot analysis of Ptpn2 protein levels (Fig. 2A), and the effects of *Ptpn2* knockout on the IFNγ response were examined. Nontargeting control (sgNTC) and *Ptpn2* knockout (sgPtpn2) B16F10 cell lines were treated with 100 ng/mL IFNγ for 72 hours and the expression of a panel of IFNγ response genes, including the T-cell chemokines *Cxcl11* and *Ccl5* (Fig. 2B), the signaling pathway genes *Stat1*, *Stat2*, *Stat3*, and *Irf1*, the antigen-presenting pathway genes *Tap1* and *Pd-l1*, and the cell-cycle regulator *Casp8,* was measured by qRT-PCR analysis (Supplementary Fig. S1A). Expression of these genes was virtually undetectable in control or *Ptpn*2 knockout cells in the absence of IFNγ treatment. Notably, however, while IFNγ induced modest expression of some IFNγ response genes in control B16F10 cells, expression of all genes examined was dramatically upregulated in IFNγ-treated *Ptpn2* knockout cells compared with control B16F10 cells treated with IFNγ (Fig. 2B; Supplementary Fig. S1A), demonstrating that *Ptpn2* deletion had restored and/or amplified the IFNγ response. To confirm that the *Ptpn2* knockout acted through effects on the Jak1/Stat1 signaling pathway, we examined expression of total Stat1 and phosphorylated (active) Stat1 protein levels by Western blotting. Indeed, IFNγ treatment of both control and *Ptpn2* knockout cells increased the level of total Stat1 and induced the expression of phosphorylated Stat1, which was absent in the untreated cells (Fig. 2C and D, left four lanes: Ctrl, Ctrl + IFNγ, sgPtpn2-3, sgPtpn2-3 + IFNγ in each panel). Moreover, Stat1 phosphorylation was induced to a greater extent in *Ptpn2* knockout cells than in control cells. We next assessed the effect of *Ptpn2* knockout on B16F10 cell proliferation. TNFα is reported to synergize with IFNγ to induce Jak1/Stat1-dependent tumor cell death and recruit cytolytic T cells to the tumor microenvironment (17, 32). Control and *Ptpn2* knockout cells were incubated for 72 hours in the presence of 100 ng/mL IFNγ alone or in combination with 20 ng/mL TNFα. As reported, proliferation of control B16F10 cells was inhibited by the combination of IFNγ + TNFα but not by IFNγ treatment alone; in contrast, *Ptpn2* knockout restored the ability of IFNγ to inhibit cell proliferation in the absence of TNFα (Supplementary Fig. S1B). Collectively, these results demonstrate that *Ptpn2* deletion sensitizes B16F10 cells to IFNγ treatment, consistent with the previously reported phenotype of *Ptpn2*-null B16F10 cells (17).

### PTPN2 Inhibitors Sensitize B16F10 Melanoma Cells to IFNγ Treatment

Having established that *Ptpn2* gene knockout sensitizes melanoma cells to IFNγ treatment, we next asked whether the small-molecule Ptpn2 inhibitors (PTP 1 through PTP 10) could have the same effect. Incubation of B16F10 cells with up to 30 μmol/L of the Ptpn2 inhibitors for 24 hours with PBS had no effect on cell viability, indicating that they were not inherently cytotoxic. However, cell proliferation was decreased when incubated with 100 ng/mL IFNγ and the inhibitors, an effect similar to the *Ptpn2* knockout cells (Supplementary Fig. S1C), suggesting that the inhibitors abolished Ptpn2 activity.

To examine the effects of the Ptpn2 inhibitors on the IFNγ response, B16F10 cells were incubated for 72 hours with vehicle (DMSO) or 30 μmol/L of each inhibitor in the presence of 100 ng/mL IFNγ. qRT-PCR analysis indicated that, as observed with *Ptpn2* gene knockout cells, expression of *Cxcl11* and *Ccl5* was undetectable in control or Ptpn2 inhibitor–treated cells in the absence of IFNγ. However, while IFNγ treatment alone induced a very low level of *Cxcl11* and *Ccl5* mRNA expression, PTP 5, 6, 7, and 9 treatment significantly increased *Cxcl11* in IFNγ-treated cells; only PTP 9 significantly upregulated both *Cxcl11* and *Ccl5* under the IFNγ stimulation condition (Fig. 2E). Similarly, only PTP 9 induced the highest both Cxcl11 and Ccl5 under the IFNγ and TNFα stimulation (Supplementary Fig. S1D). Meanwhile, we also assessed the effects of PTP 5, 6, 7, PTP 9 on Stat1 and phosphorylated Stat1 in B16F10 cells by preincubating the cells with DMSO or inhibitors for 12 hours followed by incubation with IFNγ for an additional 24 hours (Fig. 2 and D, right four lanes: PTP5, 6, 7, or 9 + IFNγ in each panel). Phosphorylated Stat1 was undetectable in cells treated with Ptpn2 inhibitors alone and was expressed at low levels in cells treated with IFNγ alone. It is noteworthy that Stat1 phosphorylation was markedly upregulated by cotreatment with Ptpn2 inhibitors and IFNγ. Moreover, proliferation assays confirmed that treatment with the Ptpn2 inhibitors further suppressed B16F10 cell proliferation in the presence of IFNγ (Supplementary Fig. S1C). On the basis of these results, we selected PTP 9 to further validate the inhibitors effect *in vivo*. To make sure the inhibitor is nontoxic to normal cells, we incubated PTP 9 with normal colon epithelial cell CCD 841 CoN or healthy donors’ primary T cells for 48 hours. PTP 9 did not affect the normal cells viability at the concentration up to 50 μmol/L (Supplementary Fig. S1E). Collectively, these results indicate that Ptpn2 inhibitors, particularly PTP 9, mimicked *Ptpn2* knockout phenotype in sensitizing B16F10 cells to IFNγ treatment *in vitro*.

### PTPN2 Inhibitor 9 Sensitizes Melanoma Tumors to PD-1 Antibody Treatment *In Vivo*

**FIGURE 3:**
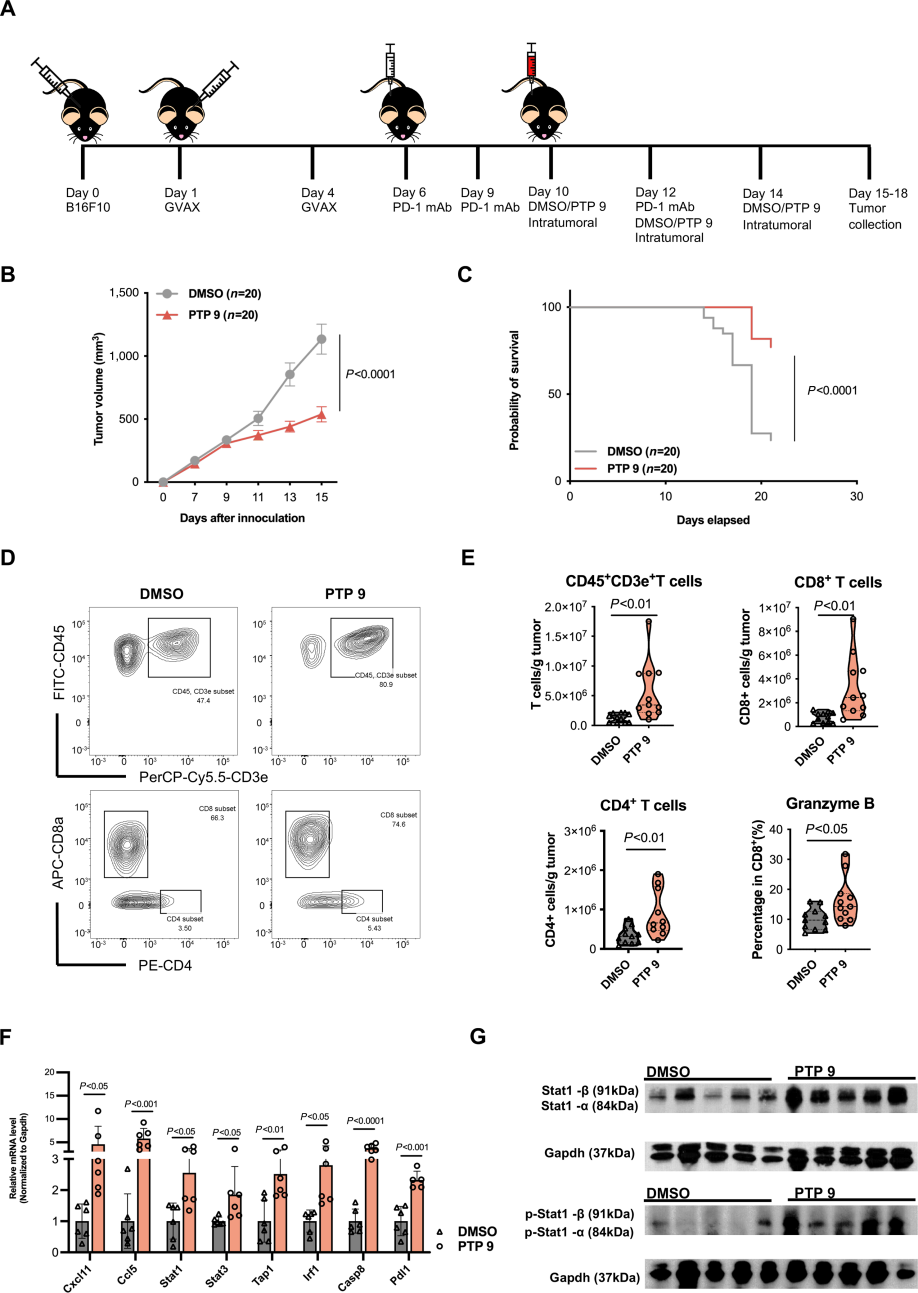
Anti-PD-1 and PTPN2 inhibitor 9 acts synergistically to inhibit B16F10 melanoma growth and promote tumor infiltration by T cells *in vivo*. **A,** Experimental design to determine the activity of PTP 9 in ati-PD-1 therapy. B16F10 melanoma cells were injected subcutaneously into C57BL/6J wildtype mice (5 × 10^5^ per mouse). Irradiated B16 GMCSF cells (GVAX cells) were subcutaneously injected (1 × 10^6^ each time per mouse) to the opposite flanks on days 1 and 4 to elicit anti-B16 immune responses due to B16 cells’ poor immunogenicity. Anti-PD-1 mAb (200 μg each time per mouse, 10 mg/kg) were injected intraperitoneally on days 6, 9, and 12. PTP 9 (50 mg/kg) or DMSO (same volume as PTP 9) was intratumorally injected on days 10, 12, and 14. Tumors were collected from day 15 to day 18 for further analyses. **B,** Tumor growth in C57BL/6 mice under anti-PD-1 immunotherapy and treated with DMSO/or PTP 9. Data are the mean ± SEM of the indicated total number of mice per group; representative of three independent experiments. Statistical analyses were performed by two-way ANOVA. **C,** Kaplan–Meier survival curves for mice treated with DMSO or PTP 9. Differences between survival curves were analyzed by using the Mantel–Cox test. **D,** Representative FACS density plots for T cells (CD45^+^ CD3e^+^), CD8^+^ T cells, and CD4^+^ T cells. Tumor-infiltrating cells were analyzed using the gating strategies described in Supplementary Fig S2B. **E,** FACS analysis of tumor-infiltrating immune cells isolated from the B16 tumors in the two groups. Data are presented by violin plot of *n* = 11. Each point represents an individual mouse. *P* values were calculated by Student *t* test. **F,** qRT-PCR analysis of tumor expression of the indicated mRNAs on day 16 in mice treated as described in **A**. Data are the mean ± SD of *n* = 6. *P* values were calculated by Student *t* test. Each point represents an individual mouse. **G,** Western blot analysis of Stat1 and phosphorylated Stat1 in tumors excised on day 16 from mice treated as described in **A**.

We next investigated whether the effect of Ptpn2 inhibitors observed *in vitro* can be translated to *in vivo* models. We examined the ability of the inhibitors to sensitize melanoma to ICI treatment, specifically anti-PD-1 therapy. For this, we employed a syngeneic mouse model in which B16F10 cells were injected subcutaneously into C57BL/6 mice on day 0, followed by subcutaneous injection of irradiated GM-CSF-secreting B16F10 cells (GVAX) into the opposite flank on days 1 and 4, intraperitoneal injection of PD-1 antibody on days 6, 9, and 12, and intratumoral injections of PTP 9 or the same volume of DMSO on days 10, 12, and 14 (Fig. 3A). A reduction in tumor growth was apparent after the injection of PTP 9 on day 10 and growth inhibition became more pronounced after the day 12 and day 14 injections (Fig. 3B; Supplementary Fig. S2A). Consistent with this finding, treatment with anti-PD-1 and PTP 9 significantly prolonged the survival of B16F10 tumor-bearing mice compared with mice treated with anti-PD-1 alone (Fig. 3C). These results demonstrate that the small-molecule Ptpn2 inhibitor PTP 9 effectively sensitizes B16F10 melanoma to ICI therapy *in vivo*.

### PTPN2 Inhibitor 9 Promotes Anti-PD-1 Efficacy by Enhancing Recruitment of CD8^+^ T Cells to the Tumor

To assess how PTP 9 might affect the antitumor immune response, groups of B16F10 tumor-bearing mice treated as described in the previous section were sacrificed from days 15 to 18, and the tumors were randomly selected for flow cytometry, Western blot analysis, or qRT-PCR analysis. Quantification of tumor-infiltrating T cells by flow cytometry showed that the number of total T cells (CD45^+^ CD3e^+^), CD4^+^ T cells and cytotoxic CD8^+^ T cells, including those producing the cytolytic protease granzyme B, were significantly increased in tumors from mice treated with PTP 9 compared with those treated with the DMSO (Fig. 3D and E). Of note, the observed increase in abundance of tumor-infiltrating granzyme B-expressing CD8^+^ T cells is consistent with the phenotype previously reported for *Ptpn2*-null B16F10 tumors (17).

We also examined tumor expression of a number of IFNγ response genes by qRT-PCR analysis. Consistent with the *in vitro* cell experiments, we observed that PTP 9 and anti-PD-1 cotreatment induced a significant increase in tumor expression of *Cxcl11, Ccl5*, *Stat1*, *Stat3*, *Tap1*, *Irf1*, *Casp8, and Pdl1,* compared with anti-PD-1 treatment alone (Fig. 3F). Furthermore, Western blot analysis showed a marked upregulation of Stat1, particularly the active phosphorylated form, in tumors from mice treated with anti-PD-1 and PTP 9 compared with anti-PD-1 plus intratumoral DMSO treatment (Fig. 3G), consistent with the *in vitro* observations that small-molecule Ptpn2 inhibition or *Ptpn2* knockout restored responsiveness of the Stat1 pathway to IFNγ in B16F10 cells. Thus, inhibition of Ptpn2 by the small-molecule PTP 9 sensitizes melanoma tumors to anti-PD-1 therapy *in vivo*, at least in part, by restoring the ability to respond to IFNγ, thereby increasing T-cell chemokine expression and recruitment of cytolytic T cells to the tumor microenvironment.

### PTPN2 Inhibitor 9 Sensitizes Mouse and Human Colorectal and Lung Cancer Cells to IFNγ Treatment

**FIGURE 4:**
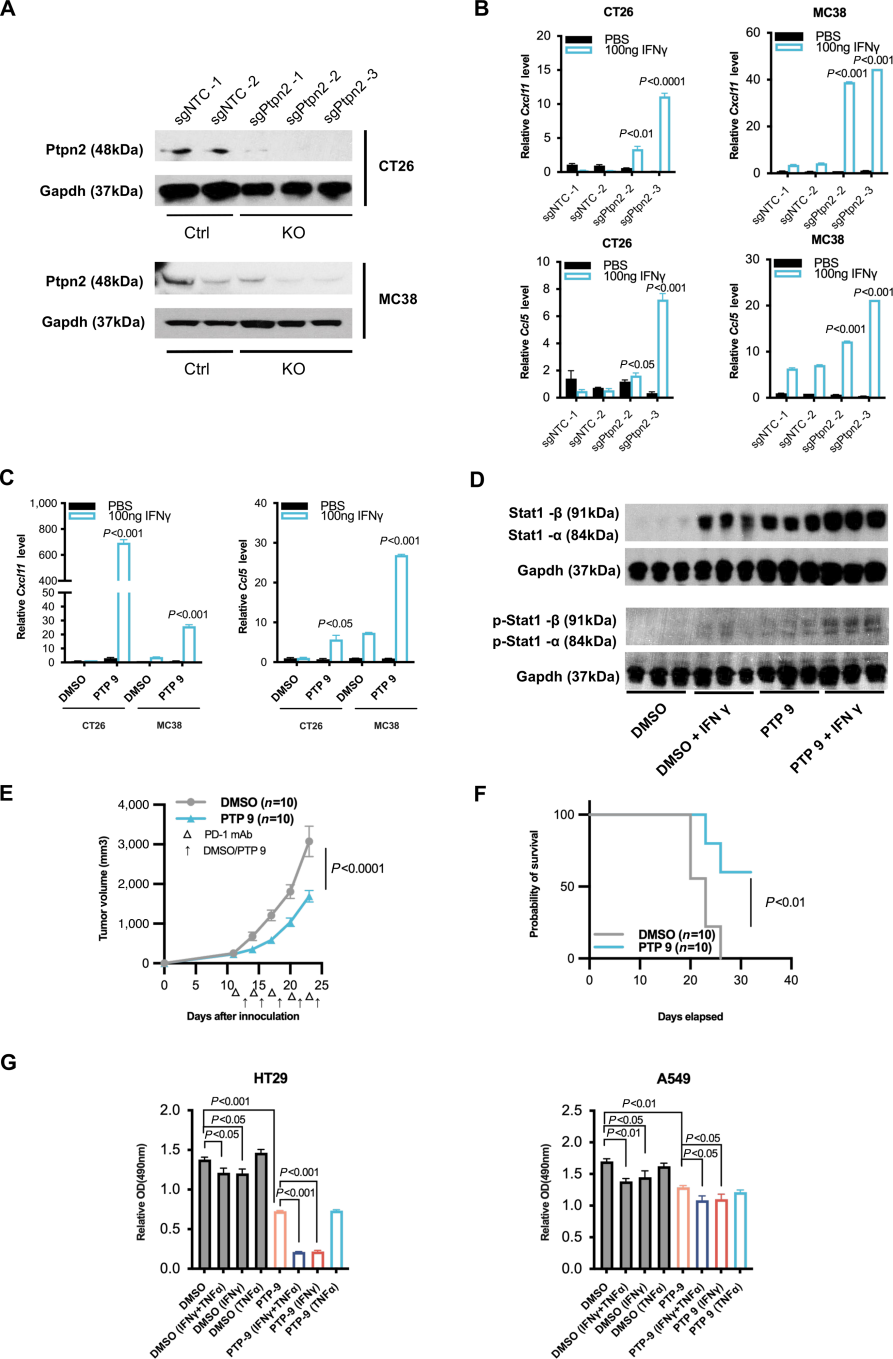
PTPN2 inhibitor 9 treatment or *Ptpn2* knockout sensitizes mouse and human colon and lung cancer cells to IFNγ treatment. **A,** Western blot analysis of Ptpn2 expression in control (sgNTC) and *Ptpn2* knockout (sgPtpn2) CT26 and MC38 murine colon carcinoma cells. **B,** qRT-PCR of *Cxcl11* and *Ccl5* mRNA in control and *Ptpn2* knockout CT26 and MC38 cell treated for 24 hours with PBS or 100 ng/mL IFNγ. *P* values compare *Ptpn2* knockout with the control both under IFNγ treatment by Student *t* test. Data are the mean ± SEM of *n* = 3. **C,** qRT-PCR of *Cxcl* and *Ccl5* mRNA in CT26 and MC38 cells incubated for 24 hours with PBS or 100 ng/mL IFNγ, together with DMSO or 30 μmol/L of PTP 9. *P* values compare the inhibitors with the DMSO control under IFNγ treatment by Student *t* test. Data are the mean ± SEM of *n* = 3. **D,** Western blot analysis of Stat1 and phosphorylated-Stat1 in CT26 and MC38 cells treated with DMSO or PTP 9, together with either PBS or IFNγ 100 ng/mL. **E,** CT26 tumor growth in Balb/c mice under anti-PD-1 immunotherapy and intratumorally treated with DMSO or PTP 9. CT26 cells were injected subcutaneously into one flank of Balb/c wildtype mice (2 × 10^6^ per mouse). Anti-PD-1 mAb (200 μg each time per mouse, 10 mg/kg) were injected intraperitoneally on days 11, 14, 17, 20, and 23. PTP 9 (25 mg/kg) or DMSO (same volume as PTP 9) was intratumorally injected on days 12, 15, 18, 21, and 24. Data are the mean ± SEM of *n* = 10; representative of two independent experiments. Tumor growths were compared and calculated by two-way ANOVA. **F,** Kaplan–Meier survival curves for mice treated with DMSO or PTP 9. **G,** Proliferation of the human colon carcinoma cells lines HT29 or A549 treated for 72 hours with DMSO or 30 μmol/L PTP 9 and either IFNγ 100 ng/mL and/or TNFα 20 ng/mL for 72 hours as indicated. Data are presented as the mean ± SEM of *n* = 4. *P* values were calculated by Student *t* test.

Because resistance to ICI therapy is observed in many solid tumors, we determined whether Ptpn2 inhibition sensitizes other cancers to IFNγ treatment. To address this question, we used CRISPR/Cas9 editing to generate stable control (sgNTC) and *Ptpn2* KO (sgPtpn2) CT26 and MC38 murine colon cancer cell lines. Effective depletion of *Ptpn2* in both cell lines was confirmed by Western blotting of Ptpn2 protein levels (Fig. 4A). The cells were then incubated for 72 hours with PBS or 100 ng/mL IFNγ and the expression of IFNγ response genes, Stat1 and phosphorylation of Stat1 were examined. As was observed for melanoma cells, IFNγ treatment of *Ptpn2* knockout CT26 and MC38 cells enhanced the expression of the IFNγ response genes *Cxcl1*, *Ccl5* (Fig. 4B), *Stat1*, *Stat2*, *Stat3*, *Irf1, Pd-l1, Tap1,* and *Casp8* (Supplementary Fig. S3A and S3B), compared with IFNγ-treated control cells. Western blot analysis also confirmed that both Stat1 and phosphorylated Stat1 were dramatically upregulated in IFNγ-treated *Ptpn2* knockout CT26 cells compared with control IFNγ-treated cells (Supplementary Fig. S3C). *Ptpn2* knockout sensitized both CT26 and MC38 cells proliferation to IFNγ treatment (Supplementary Fig. S3D), as reported previously (17). Importantly, we found that treatment of CT26 and MC38 cells with PTP 9 faithfully recapitulated the phenotypes obtained with cells depleted of Ptpn2 activity by *Ptpn2* knockout. Incubation of both cell lines with PTP 9 significantly increased the response to IFNγ compared with IFNγ-treated control cells, as illustrated by enhanced expression levels of *Cxcl11* and *Ccl5* (Fig. 4C), other immune-related genes (Supplementary Fig. S4A and S4B), more sensitization to IFNγ treatment in proliferating cells (Supplementary Fig. S4C), and increased expression of both Stat1 and phosphorylated Stat1 in CT26 (Fig. 4D). To determine the activity of PTP 9 *in vivo*, we analyzed PTP 9 in anti-PD-1 therapy's outcome using CT26 syngeneic mouse model (Fig. 4E and F; Supplementary Fig. S4D). Intratumoral injection of PTP 9 delayed the tumor growth of CT26 under anti-PD-1 treatment. PTP 9 also extended the treated mice life span, with more than half survived on day 30, compared with the median survival 23 days DMSO-treated control. Finally, we extended our investigation to examine the effects of PTP 9 on the IFNγ response to a human colon cancer cell line, HT29, and a human lung carcinoma cell line, A549. In agreement with the observations with murine cancer cells, the proliferation of both cell types in the presence of either IFNγ or IFNγ + TNFα was significantly inhibited by cotreatment with PTP 9 (Fig. 4G).

Collectively, these results confirm that the ability of PTP 9 to sensitize cancer cells to the effects of IFNγ and TNFα are not restricted to melanoma cells but extend to other solid tumors, many of which exhibit resistance to ICI therapy.

## Discussion

Although cells and animal models with gene knockout are essential tools for identifying potential therapeutic targets, translation of the findings to the clinic requires the design and testing of molecular targeting small-molecule or biologic agents. In this study, we sought to determine whether small-molecule inhibitors of Ptpn2 could replicate the phenotype previously reported for *Ptpn2*-null B16F10 cells and sensitize this resistant tumor to ICIs (17). We identified several inhibitors that were noncytotoxic, successfully upregulated expression of a variety of IFNγ response genes, and increased phosphorylation of Stat1, consistent with inhibition of Ptpn2 phosphatase function. Moreover, we confirmed the clinical relevance of our findings by demonstrating that the Ptpn2 inhibitor PTP 9 sensitized melanoma tumors to anti-PD-1 antibody treatment in a mouse melanoma model, in part by promoting chemokine secretion and recruitment of CD8^+^ granzyme B-producing T cells to the tumor microenvironment. These results are consistent with the previously demonstrated phenotype of *Ptpn2*-null B16F10 cells (17) and further show that small-molecule inhibitors of Ptpn2 may have clinical utility for the treatment of ICI-refractory tumors.

Our work in the current study was focused on melanoma and colorectal cancer; however, Ptpn2 has been implicated as an important regulator of a variety of cancers. Wu and colleagues examined glioma patient datasets from The Cancer Genome Atlas and showed that PTPN2 mRNA levels are upregulated in parallel with advancing tumor grade. Furthermore, PTPN2 deletion in human glioblastoma T98G cells was observed to prevent colony formation and induce apoptosis, demonstrating that PTPN2 was required for glioma growth (33). Macrophage-specific *Ptpn2* deletion is known to protect against colitis-associated tumor formation in mice in an inflammasome and IL1β-dependent manner (34, 35). Kleppe and colleagues demonstrated that PTPN2 regulates T-cell proliferation *via* JAK/STAT signaling in patients with T-cell acute lymphoblastic leukemia (T-ALL), and they hypothesized that PTPN2 expression correlates with tumor response to the tyrosine kinase inhibitor imatinib (11, 12, 36). Genome-wide CRISPR screens of a broad panel of mouse cancer cell lines, including B16F10 and MC38, have shown that that *Ptpn2* and other negative regulators of the IFNγ response are involved in immune evasion by multiple cancer types (37). PTPN2 has also been found to be a tumor suppressor in breast cancer and is frequently downregulated in multiple subtypes, including triple-negative breast cancer (38, 39). Furthermore, loss of PTPN2 in patients with triple-negative breast cancer was associated with poor response to tamoxifen (40, 41) demonstrating the relevance of this phosphatase to the response of human cancer to chemotherapy in addition to immunotherapy. Collectively, these findings and the results of the current study suggest an important role for PTPN2 in many cancer types, with potentially oncogenic and tumor suppressor functions. The development of small-molecule PTPN2 inhibitors such as those described here will enable further study of the mechanism by which PTPN2 inhibition sensitizes resistant tumors to immunotherapy beyond melanoma and colorectal cancer.

Despite the technical and delivery methodology issues, intratumor administration of compounds can yield high therapeutic index with low toxicity and is feasible in multiple organs by direct or image-guided injections (42, 43). In addition, the administered agents can have immediate access to tumor-draining lymph nodes, which offers great advantages to initiate an antitumor immune response (44). For example, CpG oligonucleotide TLR9 agonists were locally injected to pathologic lymph nodes in patients with follicular lymphoma following low-dose local irradiation and showed a higher tumor regression rate (45); intratumoral administration of SD-101 was safely combined with anti-PD-1 mAb (pembrolizumab) to elicit an antitumor response in patients refractory to anti-PD-1 monotherapy (46); intratumoral injections of GMCSF-encoding oncolytic virus, talimogene laherparepvec (genetically modified herpes simplex virus-1), has been approved by FDA to treat melanoma metastases in patients with stage IIIB–IVM1a (EMA) or stage IIIB–IVM1c melanoma (47). We anticipate that with additional optimization and further evaluation of their target specificity, these inhibitors could be promising lead compounds to establish PTPN2 inhibitors as immunotherapy-sensitizing agents.

## Supplementary Material

Figure S1 Supplementary Figure S1

Figure S2 Supplementary Figure S2

Figure S3 Supplementary Figure S3

Figure S4 Supplementary Figure S4

Supplementary Data SD1 Compounds design, synthesis, and characterization Table S1. Calculated physicochemical properties of Ptpn2 inhibitors.
Table S2. CRISPR target guide sequence to generate stable cell lines.
Table S3. The primers used for quantitative RT-PCR.
Figures S1 - S10. Analytical spectra for Ptpn2 inhibitors
