# Supplementary material for: Small-molecule PTPN2 Inhibitors Sensitize Resistant Melanoma to Anti-PD-1 Immunotherapy
Source: Cancer Res Commun. 2023 Jan 24;3(1):119–29. doi: 10.1158/2767-9764.CRC-21-0186 (PMC10035454; doi:10.1158/2767-9764.CRC-21-0186)
Supplement: Figure S1 — Supplementary Figure S1 [file crc-21-0186-s01.pptx]

## Slide 1
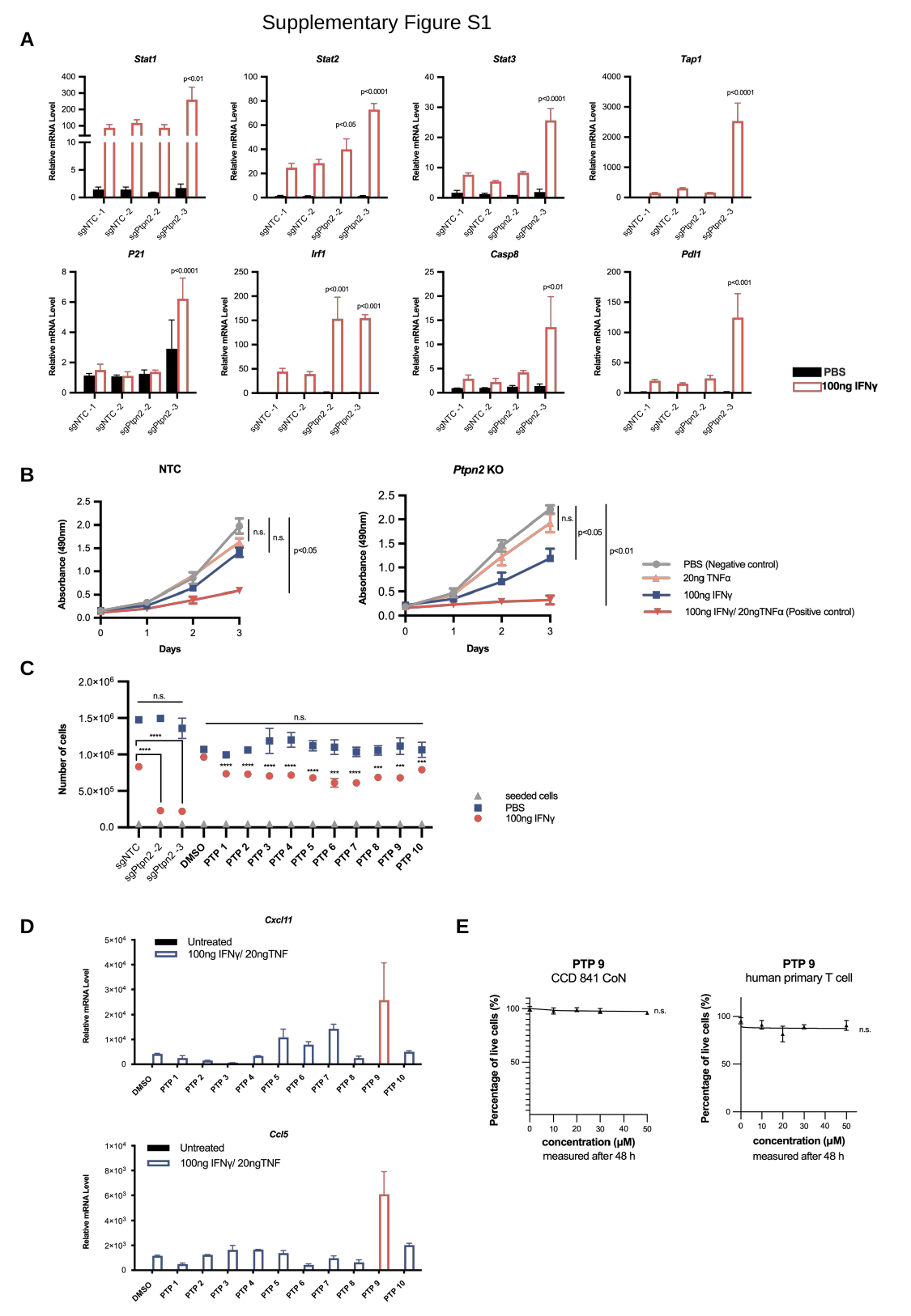

Supplementary Figure S1
A
B
C
E
D

## Slide 2
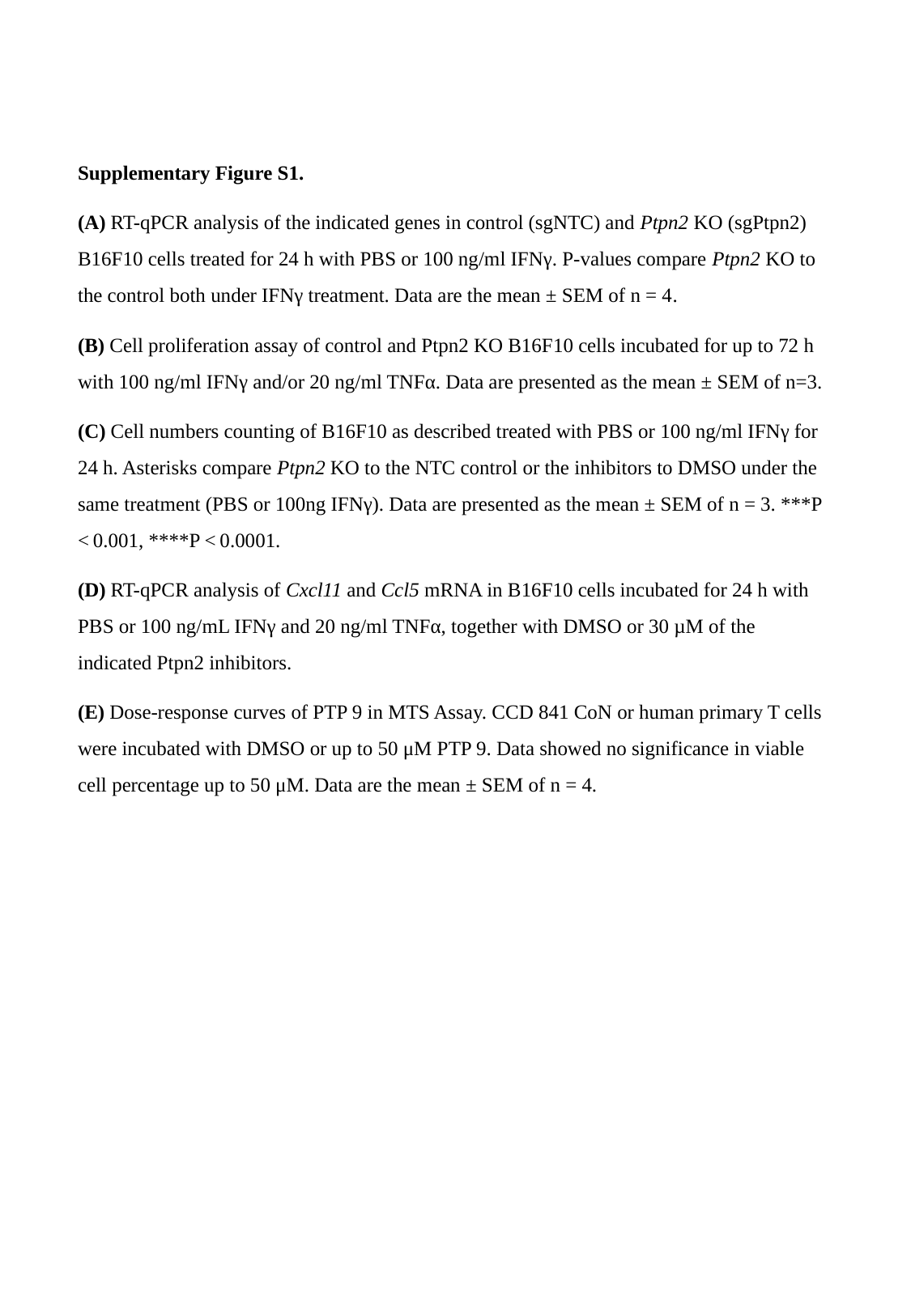

Supplementary Figure S1.
(A) RT-qPCR analysis of the indicated genes in control (sgNTC) and Ptpn2 KO (sgPtpn2) B16F10 cells treated for 24 h with PBS or 100 ng/ml IFNγ. P-values compare Ptpn2 KO to the control both under IFNγ treatment. Data are the mean ± SEM of n = 4.
(B) Cell proliferation assay of control and Ptpn2 KO B16F10 cells incubated for up to 72 h with 100 ng/ml IFNγ and/or 20 ng/ml TNFα. Data are presented as the mean ± SEM of n=3.
(C) Cell numbers counting of B16F10 as described treated with PBS or 100 ng/ml IFNγ for 24 h. Asterisks compare Ptpn2 KO to the NTC control or the inhibitors to DMSO under the same treatment (PBS or 100ng IFNγ). Data are presented as the mean ± SEM of n = 3. ***P < 0.001, ****P < 0.0001.
(D) RT-qPCR analysis of Cxcl11 and Ccl5 mRNA in B16F10 cells incubated for 24 h with PBS or 100 ng/mL IFNγ and 20 ng/ml TNFα, together with DMSO or 30 µM of the indicated Ptpn2 inhibitors.
(E) Dose-response curves of PTP 9 in MTS Assay. CCD 841 CoN or human primary T cells were incubated with DMSO or up to 50 μM PTP 9. Data showed no significance in viable cell percentage up to 50 μM. Data are the mean ± SEM of n = 4.
