# Supplementary material for: Small-molecule PTPN2 Inhibitors Sensitize Resistant Melanoma to Anti-PD-1 Immunotherapy
Source: Cancer Res Commun. 2023 Jan 24;3(1):119–29. doi: 10.1158/2767-9764.CRC-21-0186 (PMC10035454; doi:10.1158/2767-9764.CRC-21-0186)
Supplement: Figure S2 — Supplementary Figure S2 [file crc-21-0186-s02.pptx]

## Slide 1
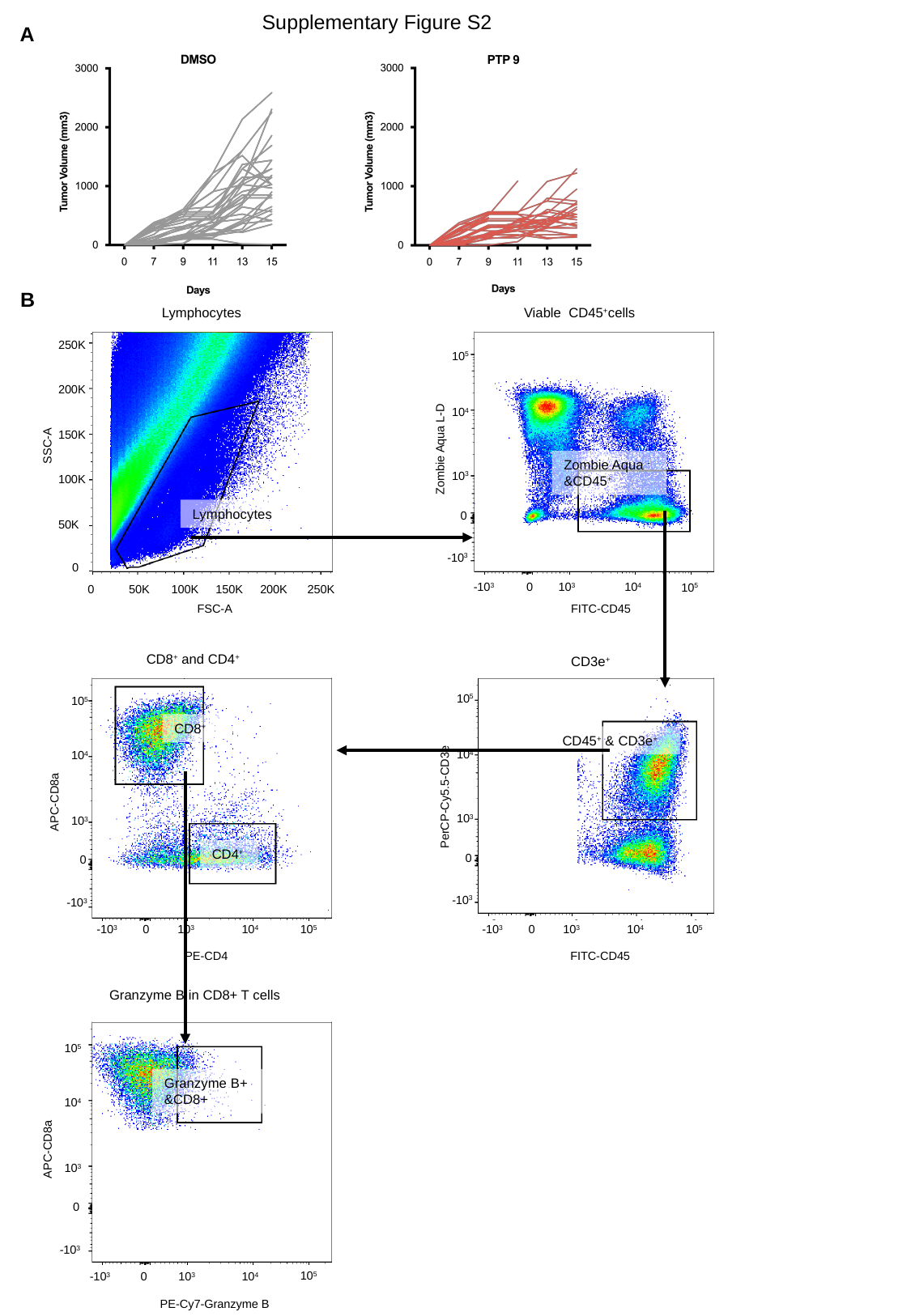

Supplementary Figure S2
A
B
Lymphocytes
Viable CD45+cells
250K
105
200K
104
Zombie Aqua L-D
SSC-A
150K
Zombie Aqua - &CD45+
103
100K
Lymphocytes
0
50K
-103
0
-103
0
103
104
105
200K
150K
100K
0
50K
250K
FSC-A
FITC-CD45
CD8+ and CD4+
CD3e+
105
105
CD8+
CD45+ & CD3e+
104
104
PerCP-Cy5.5-CD3e
APC-CD8a
103
103
CD4+
0
0
-103
-103
104
105
104
105
103
103
0
0
-103
-103
PE-CD4
FITC-CD45
Granzyme B in CD8+ T cells
105
Granzyme B+ &CD8+
104
APC-CD8a
103
0
-103
105
104
103
0
-103
PE-Cy7-Granzyme B

## Slide 2
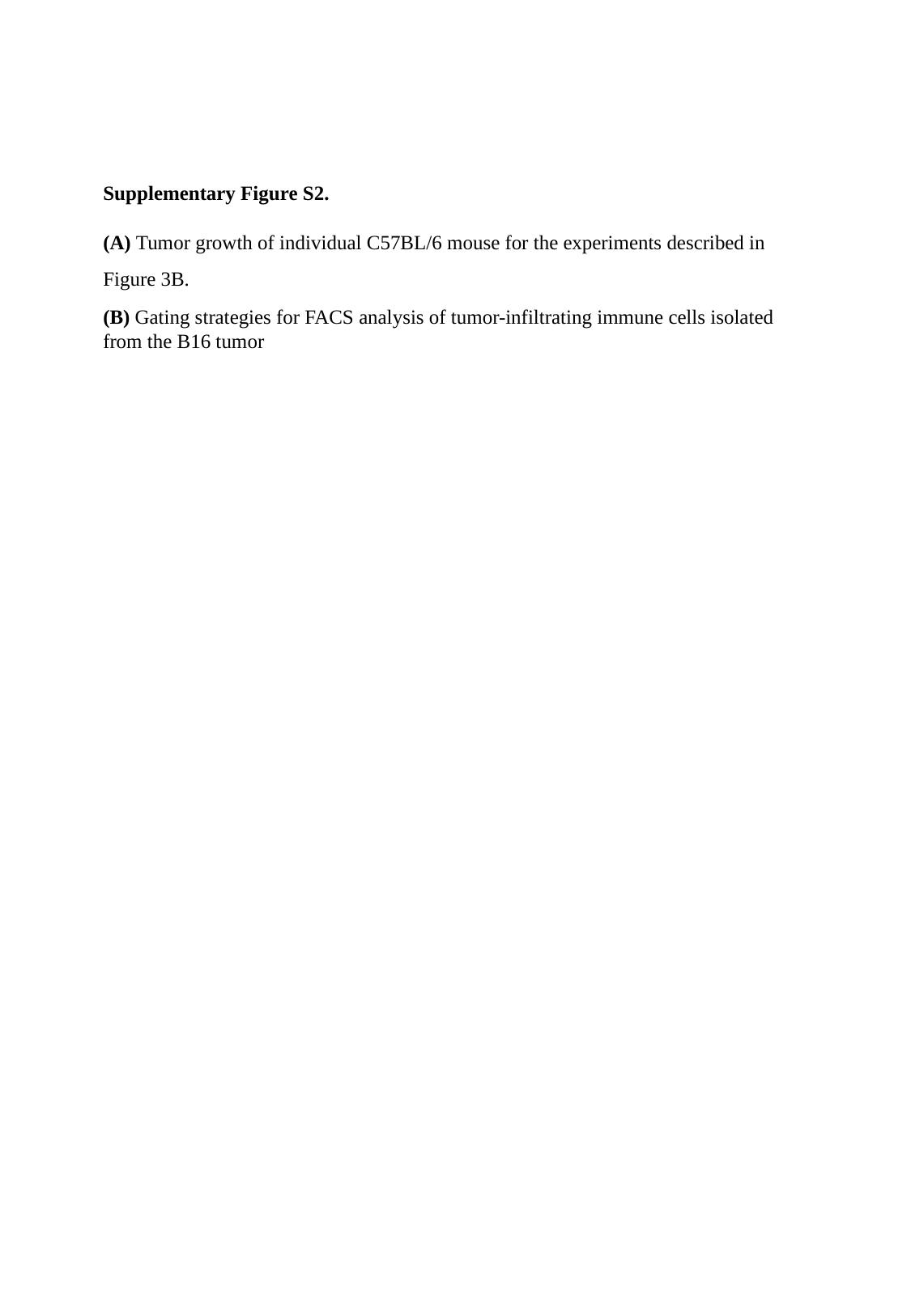

Supplementary Figure S2.
(A) Tumor growth of individual C57BL/6 mouse for the experiments described in Figure 3B.
(B) Gating strategies for FACS analysis of tumor-infiltrating immune cells isolated from the B16 tumor
