# Supplementary material for: Small-molecule PTPN2 Inhibitors Sensitize Resistant Melanoma to Anti-PD-1 Immunotherapy
Source: Cancer Res Commun. 2023 Jan 24;3(1):119–29. doi: 10.1158/2767-9764.CRC-21-0186 (PMC10035454; doi:10.1158/2767-9764.CRC-21-0186)
Supplement: Figure S3 — Supplementary Figure S3 [file crc-21-0186-s03.pptx]

## Slide 1
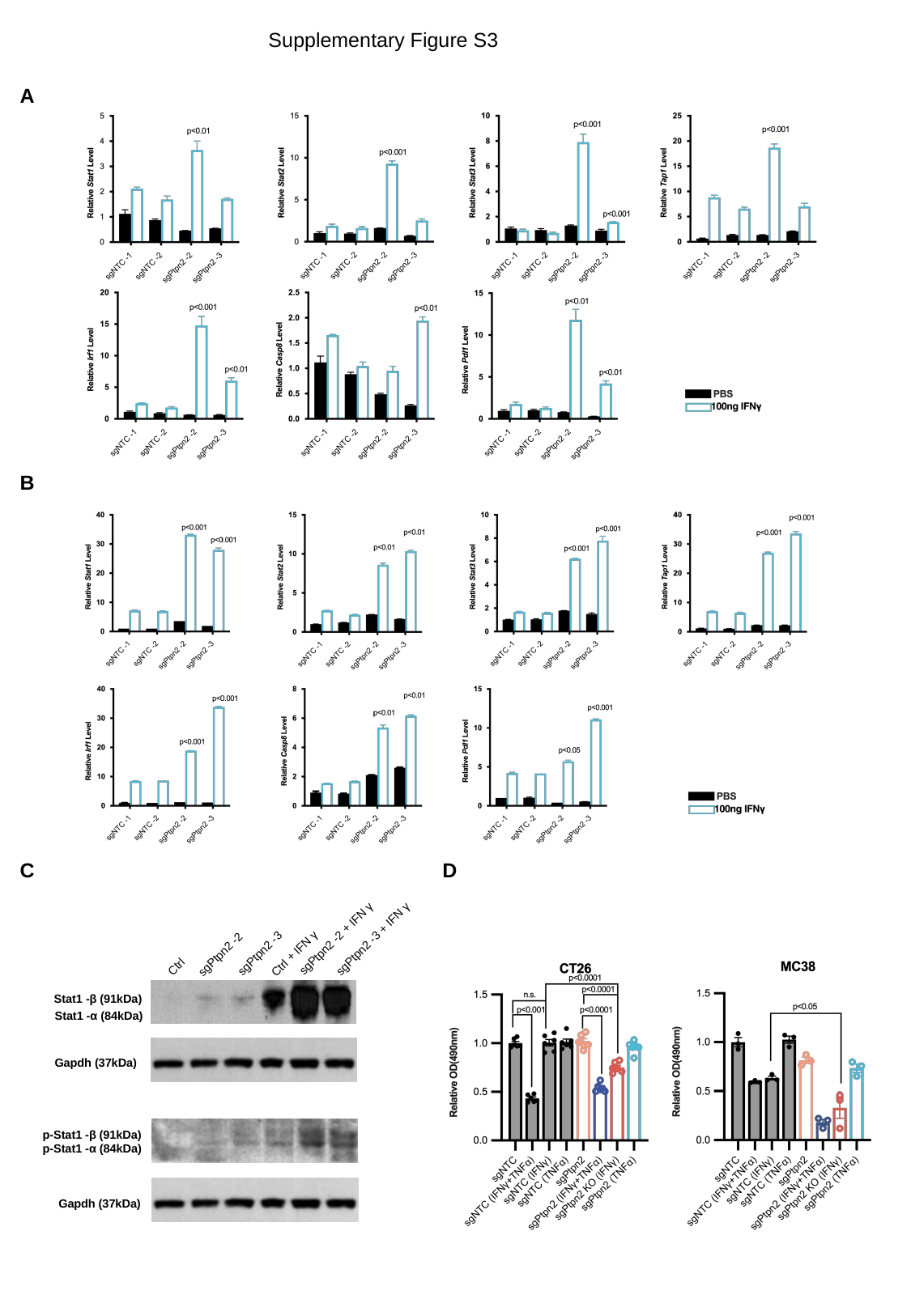

Supplementary Figure S3
A
B
C
D
sgPtpn2 -2 + IFN γ
sgPtpn2 -3 + IFN γ
sgPtpn2 -3
Ctrl
sgPtpn2 -2
Ctrl + IFN γ
Stat1 -β (91kDa)
Stat1 -α (84kDa)
Gapdh (37kDa)
p-Stat1 -β (91kDa)
p-Stat1 -α (84kDa)
Gapdh (37kDa)

## Slide 2
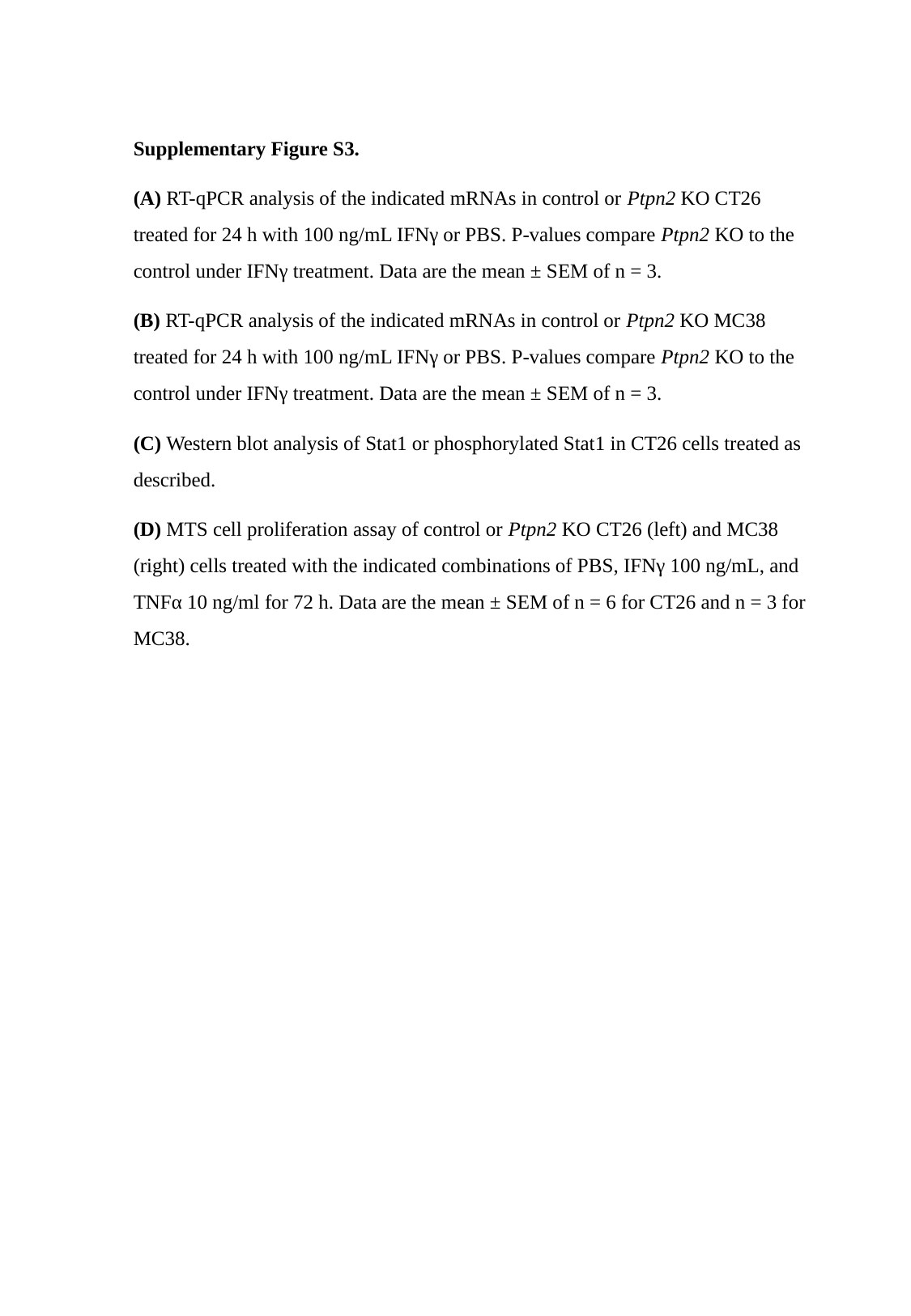

Supplementary Figure S3.
(A) RT-qPCR analysis of the indicated mRNAs in control or Ptpn2 KO CT26 treated for 24 h with 100 ng/mL IFNγ or PBS. P-values compare Ptpn2 KO to the control under IFNγ treatment. Data are the mean ± SEM of n = 3.
(B) RT-qPCR analysis of the indicated mRNAs in control or Ptpn2 KO MC38 treated for 24 h with 100 ng/mL IFNγ or PBS. P-values compare Ptpn2 KO to the control under IFNγ treatment. Data are the mean ± SEM of n = 3.
(C) Western blot analysis of Stat1 or phosphorylated Stat1 in CT26 cells treated as described.
(D) MTS cell proliferation assay of control or Ptpn2 KO CT26 (left) and MC38 (right) cells treated with the indicated combinations of PBS, IFNγ 100 ng/mL, and TNFα 10 ng/ml for 72 h. Data are the mean ± SEM of n = 6 for CT26 and n = 3 for MC38.
