# Supplementary material for: Small-molecule PTPN2 Inhibitors Sensitize Resistant Melanoma to Anti-PD-1 Immunotherapy
Source: Cancer Res Commun. 2023 Jan 24;3(1):119–29. doi: 10.1158/2767-9764.CRC-21-0186 (PMC10035454; doi:10.1158/2767-9764.CRC-21-0186)
Supplement: Figure S4 — Supplementary Figure S4 [file crc-21-0186-s04.pptx]

## Slide 1
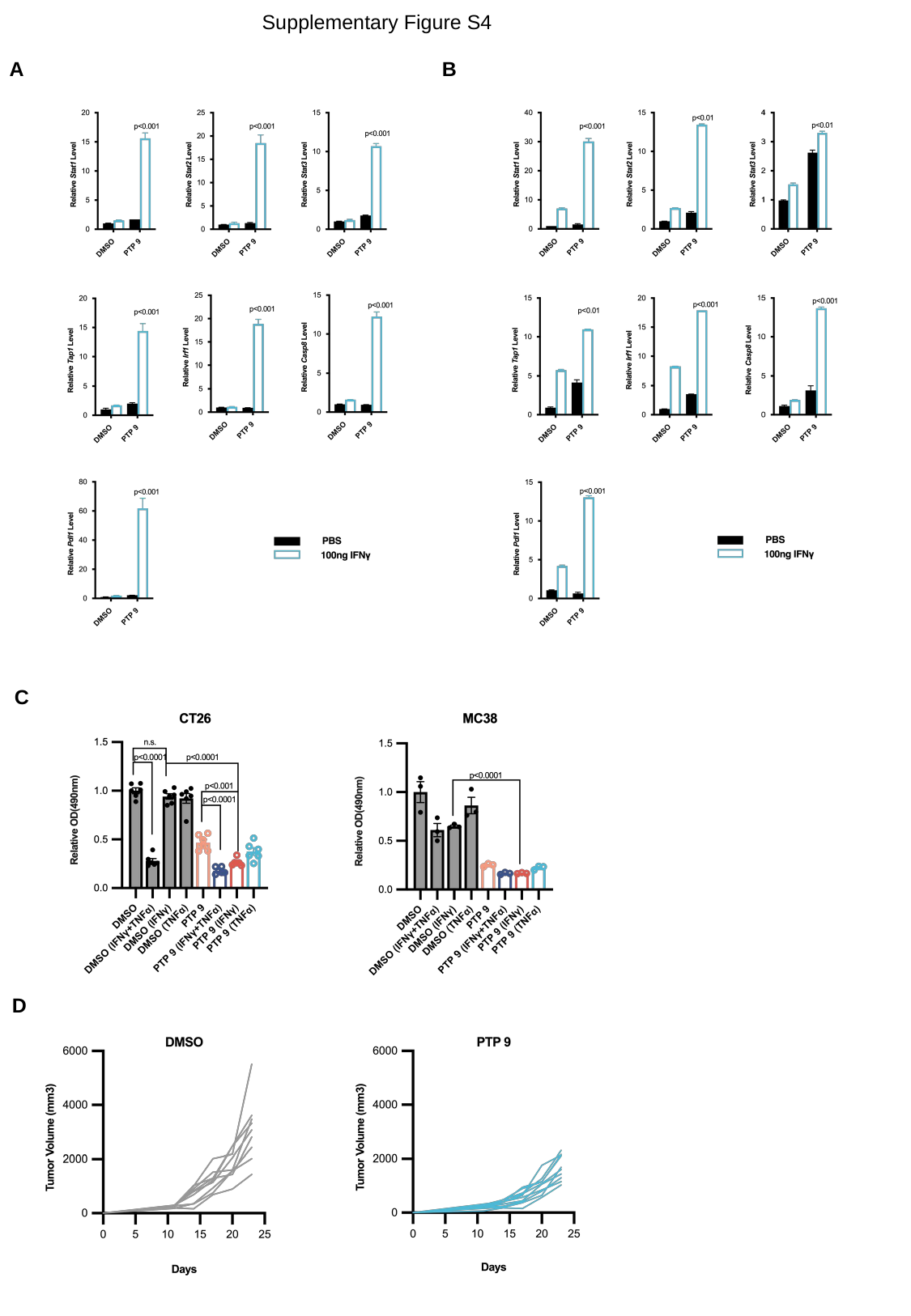

Supplementary Figure S4
A
B
C
D

## Slide 2
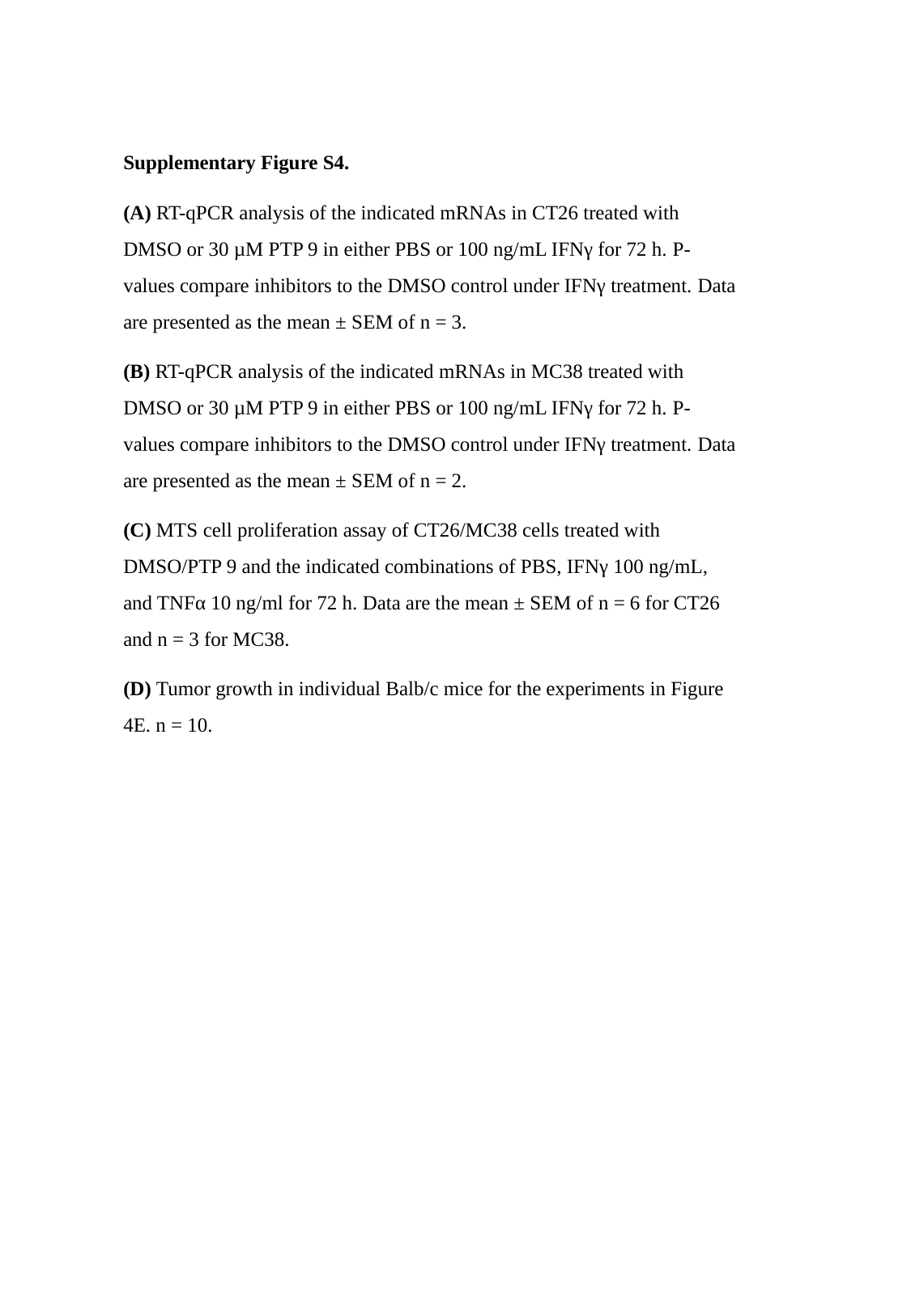

Supplementary Figure S4.
(A) RT-qPCR analysis of the indicated mRNAs in CT26 treated with DMSO or 30 µM PTP 9 in either PBS or 100 ng/mL IFNγ for 72 h. P-values compare inhibitors to the DMSO control under IFNγ treatment. Data are presented as the mean ± SEM of n = 3.
(B) RT-qPCR analysis of the indicated mRNAs in MC38 treated with DMSO or 30 µM PTP 9 in either PBS or 100 ng/mL IFNγ for 72 h. P-values compare inhibitors to the DMSO control under IFNγ treatment. Data are presented as the mean ± SEM of n = 2.
(C) MTS cell proliferation assay of CT26/MC38 cells treated with DMSO/PTP 9 and the indicated combinations of PBS, IFNγ 100 ng/mL, and TNFα 10 ng/ml for 72 h. Data are the mean ± SEM of n = 6 for CT26 and n = 3 for MC38.
(D) Tumor growth in individual Balb/c mice for the experiments in Figure 4E. n = 10.
