# Supplementary material for: Small-molecule PTPN2 Inhibitors Sensitize Resistant Melanoma to Anti-PD-1 Immunotherapy
Source: Cancer Res Commun. 2023 Jan 24;3(1):119–29. doi: 10.1158/2767-9764.CRC-21-0186 (PMC10035454; doi:10.1158/2767-9764.CRC-21-0186)
Supplement: Supplementary Data SD1 — Compounds design, synthesis, and characterization Table S1. Calculated physicochemical properties of Ptpn2 inhibitors. Table S2. CRISPR target guide sequence to generate stable cell lines. Table S3. The primers used for quantitative RT-PCR. Figures S1 - S10. Analytical spectra for Ptpn2 inhibitors [file crc-21-0186-s05.pdf]

Supplementary Information

Table of Contents

Experimental Procedures

In silico modeling and structure-based design of Ptpn2 inhibitors……………………………….2

Synthetic Methods…………………………………………………………………….....2-8

General Procedures, Materials & Instrumentation………………………………………………2-4

Experimental procedures and characterization data of Ptpn2 inhibitors.……………………...4-8

Tables and Figures

Table S1. Calculated physicochemical properties of Ptpn2 inhibitors…………………………….9

Table S2. CRISPR target guide sequence to generate stable knockout cell lines…………………9

Table S3. Primers for RT-PCR…………………………………………………………………….9

Copies of analytical spectra for Ptpn2 inhibitors.………………..……………………….10-27

References……………………………………………………………………………..28

Experimental Procedures

In silico modeling and structure-based design of Ptpn2 inhibitors

Molecular docking with the Schrödinger software suite was performed in using the X-ray crystal structure of the catalytic PTP domain of Ptpn2 (PDB ID: 1L8K).[1] The structure was prepared using the Protein Preparation Wizard in Prime and docking was performed with Glide XP.[2-6] The docking grid was defined as a 10x10x10 Å cube centered on the coordinates x = -6.0 y = 11.0 z = -4.0. Docking poses were evaluated for interactions with both the conserved HCX_5R motif and residues at the periphery of the binding pocket, such as Tyr 48 and Gln 260. Chemical modifications were made to favor cell membrane permeability as determined by Qikprop (≥ 500 nm/s for Caco-2, MDCK models).

Synthetic Methods

General Information. All reactions were performed in flame-dried round-bottomed or modified Schlenk flasks fitted with rubber septa under a positive pressure of argon, unless otherwise noted. Air- and moisture-sensitive liquids and solutions were transferred via syringe or stainless steel cannula. Tetrahydrofuran was purified using a Pure-Solv MD-5 Solvent Purification System (Innovative Technology). All other reagents were used directly from the supplier without further purification unless otherwise noted. Organic solutions were concentrated by rotary evaporation at ~25 mbar in a water bath heated to 40 °C unless otherwise noted. Analytical thin-layer chromatography (TLC) was carried out using 0.2 mm commercial glass-coated silica gel plates (silica gel 60, F254, EMD chemical). Thin layer chromatography plates were visualized by exposure to ultraviolet light and/or exposure to iodine, or to an acidic solution of ceric ammonium molybdate, or a basic solution of potassium permanganate followed by heating on a hot plate. Gas chromatographs were measured using an Agilent 7820 GC. Mass spectra (MS) were obtained on a Karatos MS9, Autospec, or an Agilent 6150 and reported as m/z (relative intensity). Accurate masses are reported for the molecular ion $[M+D]^{+}$ or $[M+2D]^{2+}$. Nuclear magnetic resonance spectra (^{1}H-NMR and ^{13}C-NMR) were recorded with a Varian Gemini (400 MHz, ^{1}H at 400 MHz, ^{13}C at 100 MHz, 500 MHz, ^{1}H at 500 MHz, ^{13}C at 125 MHz, or 600 MHz, ^{1}H at 600 MHz, ^{13}C at 150 MHz). For $CDCl_3$ and CD_3OD solutions chemical shifts are reported as parts per million (ppm) referenced to residual protium or carbon of the solvent; $CDCl_3$ δ 77.0 ppm, CD_3OD δ 3.49 ppm. Coupling constants are reported in Hertz (Hz). Data for ^{1}H-NMR spectra are reported as follows:

chemical shift (ppm, referenced to protium; (bs = broad singlet, s = singlet, br d = broad doublet, d = doublet, t = triplet, q = quartet, dd = doublet of doublets, td = triplet of doublets, ddd = doublet of doublet of doublets, m = multiplet, integration, and coupling constants (Hz)). Purity was quantified by HPLC. HPLC was performed on an Agilent 1200 series HPLC with a Supelco Analytical Discovery® C18 (25 cm X 10 mm, 5µm) RP-HPLC.

SCHEME-1

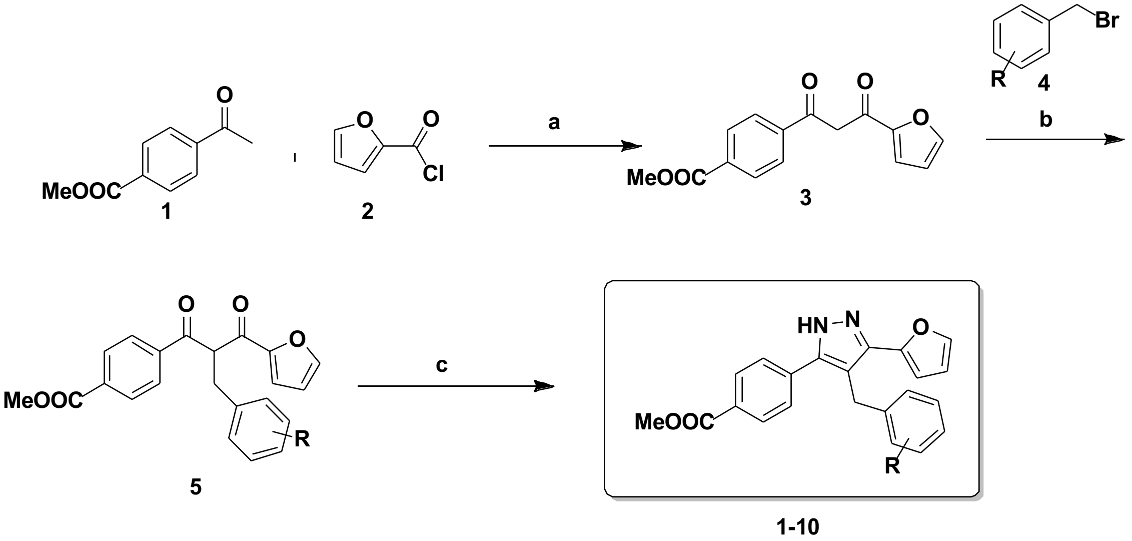

Reagents and conditions: a). LiHMDS, THF, 0 °C to RT 12 h b). K_2CO_3, NaI, acetone, reflux, 12h c). N_2H_4, EtOH, 80 °C, 2 h

Preparation of methyl 4-(3-(furan-2-yl)-3-oxopropanoyl)benzoate (3):

A suspension of compound **1** (200 mg, 0.685 mmol) in dry THF (1.0 mL) was cooled at 0 °C under Ar atmosphere. After cooling, LiHMDS (0.685 mmol) was added dropwise to the reaction mass and stirred for 30 min at 0 °C. The reaction mixture was then treated with 2-furoylchloride **2** (0.685 mmol) dissolved in THF and stirred for 12 h at room temperature. Cold ethyl acetate (150 mL) and ice-water (50 mL) were added the reaction mixture and the organic phase was separated. The aqueous phase was extracted with ethyl acetate (2 x 100 mL) and the combined extracts were

washed with water then brine and dried over $MgSO_4$. The solvents were evaporated under reduced pressure and the crude product was purified by column chromatography on silica gel (ethyl acetate:hexane, 1:9), yielding compound **3** as a white solid. (85% yield).

General procedure for the synthesis of compound (5):

A stirred suspension of methyl 4-(3-(furan-2-yl)-3-oxopropanoyl)benzoate **3** (1.0 g, 4.46 mmol), K_2CO_3 (0.67 g, 4.82 mmol) and NaI (0.73 g, 4.88 mmol) in dry acetone (15 ml) was treated with the corresponding bromo compound **4** (5.2 mmol) dropwise under N_2 atmosphere. The mixture was stirred at reflux for 12 h. The reaction was monitored by TLC and after completion of the reaction the solution was cooled to room temperature and filtered through a celite pad. The filtrate was evaporated under vacuum and the residue was purified by silica gel column chromatography (95:5, hexane:ethyl acetate) to give the pure product **4** as a colorless solid.

General procedure for the synthesis of the final compound (PTP-01 to 10):

A stirred solution of the corresponding starting material **5** (1 eq) in EtOH and was treated by the addition of N_2H_4 (1 eq)). The reaction mixture was stirred for 2 h at 80 $^{\circ}$C. After completion of the reaction as monitored by TLC, the excess of EtOH was removed under vacuum and the crude product was resuspended in water and extracted with ethyl acetate. The organic layers were combined and dried by $MgSO_4$ and filtrate was concentrated under reduced pressure. The crude product was purified by column chromatography on silica gel (ethyl acetate:hexane, 1:1) yielded compound as a white solid. (65% yield).

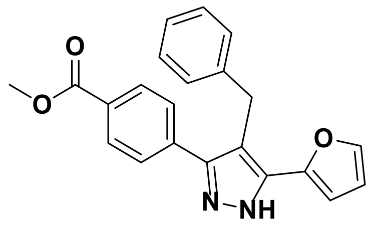

methyl 4-(4-benzyl-5-(furan-2-yl)-1*H*-pyrazol-3-yl)benzoate (1): ^{1}H-NMR (600 MHz, $CDCl_3$) δ 8.01 (d, *J* = 8.1 Hz, 2H), 7.58 (d, *J* = 8.1 Hz, 2H), 7.44 (s, 1H), 7.32 (d, *J* = 7.4 Hz, 1H), 7.29 (d, *J* = 8.5 Hz, 1H), 7.23 (t, *J* = 7.3 Hz, 1H), 7.19 (d, *J* = 7.5 Hz, 2H), 6.43 – 6.36 (m, 2H), 4.18 (s, 2H), 3.93 (s, 3H). ^{13}C-NMR (151 MHz, $CDCl_3$) δ 166.9, 154.7, 142.3, 142.3, 139.7, 139.7, 130.0, 130.0, 129.5, 129.5, 128.9, 128.9, 128.0, 128.0, 127.5, 127.5, 126.4, 112.8, 111.6, 107.7, 52.2, 29.6. HRMS (ESI): *m/z* calculated for $C_{22}H_{18}N_2O_3$ 358.1317, found 359.1388 (M+ H^+). Purity >98%.

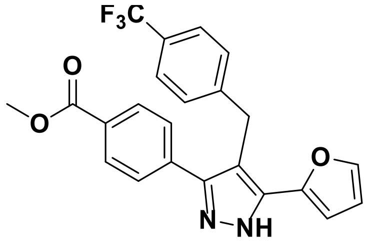

methyl 4-(5-(furan-2-yl)-4-(4-(trifluoromethyl)benzyl)-1*H*-pyrazol-3-yl)benzoate (2): ^{1}H-NMR (600 MHz, $CDCl_3$) δ 8.04 (d, *J* = 8.3 Hz, 2H), 7.55 (dd, *J* = 11.5, 8.4 Hz, 4H), 7.47 (s, 1H), 7.29 (d, *J* = 8.5 Hz, 2H), 6.45 (dd, *J* = 3.3, 1.7 Hz, 1H), 6.38 (d, *J* = 3.3 Hz, 1H), 4.24 (s, 2H), 3.94 (s, 3H). ^{13}C-NMR (151 MHz, $CDCl_3$) δ 166.7, 158.0, 144.1, 142.5, 130.2, 130.2, 129.8, 129.8, 128.8, 128.6, 128.3, 127.6, 127.6, 125.7, 125.7, 111.9, 111.9, 107.6, 52.3, 29.4. HRMS (ESI): *m/z* calculated for $C_{23}H_{17}F_3N_2O_3$ 426.1191, found 427.1263 (M+ H^+). Purity >96%.

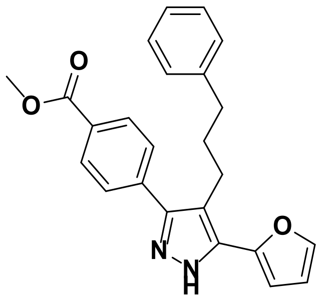

methyl 4-(5-(furan-2-yl)-4-(3-phenylpropyl)-1*H*-pyrazol-3-yl)benzoate (3): ^{1}H-NMR (600 MHz, $CDCl_3$) δ 8.08 (d, *J* = 8.1 Hz, 2H), 7.61 (d, *J* = 8.1 Hz, 2H), 7.48 (s, 1H), 7.30 (t, *J* = 5.5 Hz, 2H), 7.23 (d, *J* = 6.7 Hz, 1H), 7.15 (d, *J* = 7.3 Hz, 2H), 6.49 (s, 1H), 6.44 (s, 1H), 3.98 (s, 3H), 2.82 – 2.78 (m, 2H), 2.68 (t, *J* = 7.1 Hz, 2H), 1.94 – 1.89 (m, 2H). ^{13}C-NMR (151 MHz, MeOD) δ 166.9, 157.4, 153.7, 149.9, 142.1, 130.1, 130.1, 129.5, 128.4, 128.4, 127.6, 127.6, 126.0, 124.9,

124.2, 120.2, 115.9, 111.2, 107.0, 91.7, 52.3, 35.7, 31.8, 24.1. HRMS (ESI): *m/z* calculated for $C_{24}H_{22}N_2O_3$ 386.1630, found 387.1701 (M+ H^+). Purity >98%.

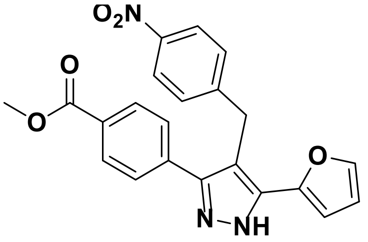

methyl 4-(5-(furan-2-yl)-4-(4-nitrobenzyl)-1*H*-pyrazol-3-yl)benzoate (4): ^{1}H-NMR (600 MHz, $CDCl_3$) δ 8.15 (d, *J* = 8.4 Hz, 2H), 8.02 (d, *J* = 8.0 Hz, 2H), 7.50 (d, *J* = 7.9 Hz, 2H), 7.43 (s, 1H), 7.32 (d, *J* = 8.3 Hz, 2H), 6.42 (d, *J* = 21.0 Hz, 2H), 4.29 (s, 2H), 3.94 (s, 3H). ^{13}C-NMR (151 MHz, $CDCl_3$) δ 166.6, 158.1, 147.8, 146.7, 142.6, 142.6, 136.5, 130.2, 130.2, 129.9, 129.9, 128.7, 128.7, 127.6, 127.6, 124.0, 124.0, 111.6, 111.4, 107.7, 52.6, 30.0. HRMS (ESI): *m/z* calculated for $C_{22}H_{17}N_3O_5$ 403.1168, found 404.1238 (M+ H^+). Purity >95%.

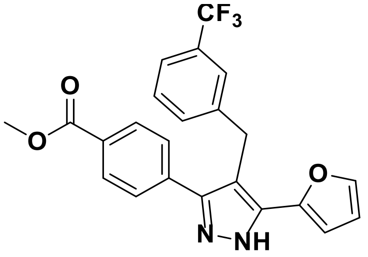

methyl 4-(5-(furan-2-yl)-4-(3-(trifluoromethyl)benzyl)-1*H*-pyrazol-3-yl)benzoate (5): ^{1}H-NMR (600 MHz, $CDCl_3$) δ 8.04 (d, *J* = 8.2 Hz, 2H), 7.54 (d, *J* = 8.2 Hz, 2H), 7.49 (d, *J* = 7.7 Hz, 1H), 7.45 (d, *J* = 5.5 Hz, 2H), 7.40 (t, *J* = 7.7 Hz, 1H), 7.33 (d, *J* = 7.7 Hz, 1H), 6.45 (dd, *J* = 3.1, 1.7 Hz, 1H), 6.40 (d, *J* = 3.3 Hz, 1H), 4.24 (s, 2H), 3.94 (s, 3H). ^{13}C-NMR (151 MHz, $CDCl_3$) δ 166.7, 142.5, 140.8, 136.3, 131.1, 131.1, 131.1, 130.9, 130.1, 130.1, 129.8, 129.8, 129.2, 129.2, 127.6, 127.6, 124.7, 123.3, 111.9, 111.7, 107.3, 52.3, 29.6. HRMS (ESI): *m/z* calculated for $C_{23}H_{17}F_3N_2O_3$ 426.1191, found 427.1261 (M+ H^+). Purity >96%.

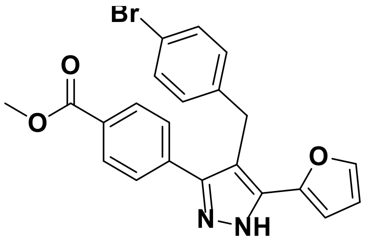

methyl 4-(4-(4-bromobenzyl)-5-(furan-2-yl)-1*H*-pyrazol-3-yl)benzoate (6): ^{1}H-NMR (600 MHz, $CDCl_3$) δ 8.04 (d, *J* = 8.3 Hz, 2H), 7.55 (d, *J* = 8.2 Hz, 2H), 7.46 (d, *J* = 1.1 Hz, 1H), 7.43 (d, *J* = 8.3 Hz, 2H), 7.06 (d, *J* = 8.3 Hz, 2H), 6.44 (dd, *J* = 3.2, 1.7 Hz, 1H), 6.38 (d, *J* = 3.3 Hz, 1H), 4.12 (s, 2H), 3.94 (s, 3H). ^{13}C-NMR (151 MHz, $CDCl_3$) δ 166.7, 144.3, 142.5, 138.8, 135.8, 131.8, 131.8, 130.1, 130.1, 130.1, 129.7, 129.7, 129.7, 129.7, 127.6, 127.6, 120.2, 112.2, 111.6, 107.8, 52.0, 29.1. HRMS (ESI): *m/z* calculated for $C_{22}H_{17}BrN_2O_3$ 436.0423, found 437.0491 (M+ H^+). Purity >96%.

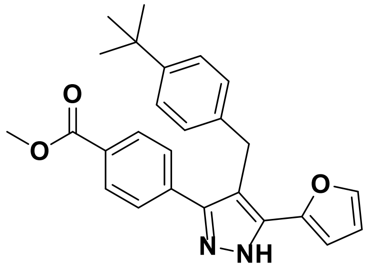

methyl 4-(4-(4-(tert-butyl)benzyl)-5-(furan-2-yl)-1*H*-pyrazol-3-yl)benzoate (7): ^{1}H-NMR (600 MHz, $CDCl_3$) δ 8.04 (d, *J* = 8.2 Hz, 2H), 7.61 (d, *J* = 8.2 Hz, 2H), 7.47 (s, 1H), 7.32 (d, *J* = 8.2 Hz, 2H), 7.11 (d, *J* = 8.1 Hz, 2H), 6.46 – 6.40 (m, 2H), 4.14 (s, 2H), 3.94 (s, 3H), 1.32 (s, 9H). ^{13}C-NMR (151 MHz, $CDCl_3$) δ 166.9, 149.0, 142.3, 136.5, 130.1, 130.1, 130.1, 129.5, 129.5, 127.6, 127.6, 127.6, 127.5, 127.5, 127.5, 125.6, 125.6, 113.2, 111.7, 107.8, 52.1, 34.3, 31.3, 31.4, 31.4, 29.1. HRMS (ESI): *m/z* calculated for $C_{26}H_{26}N_2O_3$ 414.1943, found 415.2012 (M+ H^+). Purity >98%.

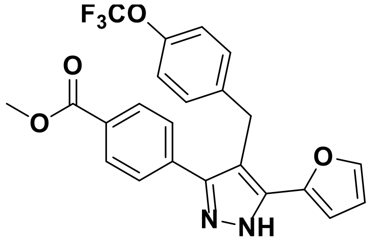

methyl 4-(5-(furan-2-yl)-4-(4-(trifluoromethoxy)benzyl)-1*H*-pyrazol-3-yl)benzoate (8): ^{1}H-NMR (600 MHz, $CDCl_3$) δ 8.04 (d, *J* = 8.1 Hz, 2H), 7.55 (d, *J* = 8.0 Hz, 2H), 7.46 (s, 1H), 7.19 (d, *J* = 8.4 Hz, 2H), 7.15 (d, *J* = 8.4 Hz, 2H), 6.47 – 6.38 (m, 2H), 4.18 (s, 2H), 3.94 (s, 3H). ^{13}C-NMR (151 MHz, $CDCl_3$) δ 166.7, 147.7, 145.4, 142.5, 138.5, 138.5, 130.1, 130.1, 130.1, 129.8, 129.8, 129.2, 129.2, 129.2, 127.6, 127.6, 127.6, 121.3, 121.3, 111.7, 107.7, 52.3, 29.1. HRMS (ESI): *m/z* calculated for $C_{23}H_{17}F_3N_2O_4$ 442.1140, found 443.1210 (M+ H^+). Purity >99%.

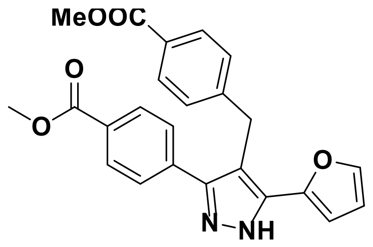

methyl 4-((5-(furan-2-yl)-3-(4-(methoxycarbonyl)phenyl)-1*H*-pyrazol-4-yl)methyl)benzoate (9): ^{1}H-NMR (600 MHz, $CDCl_3$) δ 7.97 (d, *J* = 8.1 Hz, 2H), 7.89 (d, *J* = 5.9 Hz, 1 H), 7.82 (d, *J* = 5.9 Hz, 1H), 7.53 (d, *J* = 8.1 Hz, 1H), 7.47 – 7.42 (m, 1H), 7.27 – 7.23 (m, 2H), 7.05 (d, *J* = 6.0 Hz, 1H), 6.39 (s, 1H), 6.35 – 6.31 (m, 1H), 4.77 (s, 1H), 4.23 (s, 2H), 3.92 (s, 3H), 3.89 (s, 3H). ^{13}C NMR (151 MHz, MeOD) δ 167.1, 156.0, 152.3, 146.1, 145.4, 141.1, 139.9, 133.5, 130.8, 130.1, 129.8, 129.5, 128.9, 128.6, 128.4, 128.0, 127.6, 126.5, 112.0, 111.6, 107.7, 52.3, 52.2, 29.8. HRMS (ESI): *m/z* calculated for $C_{24}H_{20}N_2O_5$ 416.1372, found 417.1445 (M+ H^+). Purity >96%.

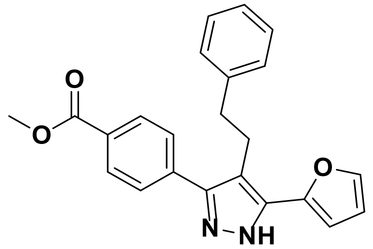

methyl 4-(5-(furan-2-yl)-4-phenethyl-1*H*-pyrazol-3-yl)benzoate (10): ^{1}H-NMR (600 MHz, $CDCl_3$) δ 8.12 (d, *J* = 8.4 Hz, 2H), 8.10 (d, *J* = 8.4 Hz, 1H), 7.86 (d, *J* = 8.2 Hz, 2H), 7.55 (d, *J* = 7.4 Hz, 2H), 7.50 (s, 1H), 6.87 (s, 1H), 7.12, (d *J* = 5.9 Hz, 1H), 6.70 (dd, *J* = 9.4, 3.2 Hz, 1H), 6.54 (s, 1H), 3.96 (s, 3H), 3.15 – 3.07 (m, 2H), 2.91 – 2.87 (m, 2H). ^{13}C-NMR (151 MHz, MeOD) δ 166.9, 160.6, 143.5, 136.9, 132.7, 130.3, 130.0, 130.0, 129.8, 129.8, 129.6, 129.6, 128.5, 128.5, 128.4, 127.8, 125.3, 125.3, 111.7, 107.0, 52.3, 36.4, 25.9. HRMS (ESI): *m/z* calculated for $C_{23}H_{20}N_2O_3$ 372.1474, found 373.1545 (M+ H^+). Purity >96%.

Table S1. Calculated physicochemical properties of Ptpn2 inhibitors.

Compound	MW (g/mol)	Rotatable Bonds	Hydrogen Bond Donors	Hydrogen Bond Acceptors	logP (octanol/ water)	Permeability (nm/s) Caco-2	Permeability (nm/s) MDCK	Solvent Accessible Surface Area ($Å^2$)	Volume ($Å^3$)	Polar Surface Area ($Å^2$)
ID_1	358.396	3	1	3	4.827	970.219	478.804	653.54	1155.719	71.522
ID_2	426.394	3	1	3	5.803	979.429	2085.459	699.48	1252.034	71.686
ID_3	386.449	5	1	3	5.703	981.599	484.877	726.458	1286.737	71.313
ID_4	403.393	4	1	4	4.115	115.35	47.918	688.842	1227.846	116.593
ID_5	426.394	3	1	3	5.434	1065.51	1561.954	659.748	1207.019	73.534
ID_6	437.292	3	1	3	5.39	969.877	1268.106	679.206	1207.538	71.631
ID_7	414.503	4	1	3	5.921	1078.143	536.623	711.392	1340.901	73.485
ID_8	442.394	4	1	3	5.545	1255.987	2010.768	642.38	1213.509	80.961
ID_9	416.432	4	1	5	4.398	257.152	113.982	726.581	1303.58	109.802
ID_10	372.423	4	1	3	5.28	962.737	474.814	692.922	1223.293	70.657

Table S2. CRISPR target guide sequence to generate stable cell lines.

CRISPR sgRNA sequences	
NTC – sg1	GCGAGGTATTCGGCTCCGCG
NTC – sg2	GCTTTCACGGAGGTTCGACG
NTC – sg3	ATGTTGCAGTTCGGCTCGAT
NTC – sg4	ACGTGTAAGGCGAACGCCTT
Ptpn2 – sg1	CCATGACTATCCTCATAGAG
Ptpn2 – sg2	TCATTCACAGAACAGAGTGA
Ptpn2 – sg3	ATGTGCACAGTACTGGCCAA

Table S3. The primers used for quantitative RT-PCR.

Primers used for quantitative RT-PCR			
Gene name	Upper primer (5'-3')	Lower primer (5'-3')	Species
Cxcl 11	GGCTGCGACAAAGTTGAAGT	CGAGCTTGCTTGGATCTGGG	Mouse
Ccl 5	GTTCCATCTCGCCATTCATGC	TAAGCAAACACAACGCAGCTC	Mouse
Tap 1	TTCACCCGCAACATATGGCT	ATGTGATGGAACCTGCTGGG	Mouse
Stat 1	GGCCTCTCATTGTCACCGAA	TACCACAGGATAGACGCCCA	Mouse
Stat 2	GTCGTCTTCAGACCCCCATC	GCCAACCAGTCCTTTGGAGA	Mouse
Stat 3	GGAACAGATGCTCACAGCCC	AGTCAGTGTCTTCTGCACGTA	Mouse
P21	TGGACAGTGAGCAGTTGCG	CGTCTCCGTGACGAAGTCAA	Mouse
Casp 8	TAGAAGGCTACCAAAGCGCA	CCCTTGTCACCGTGGGATAG	Mouse
Pd-l1	TGGTGGAGTATGGCAGCAAC	CCCAGTACACCACTAACGCA	Mouse
Irf 1	GGAGATGTTAGCCCGGACAC	AGGTAGCCCTGAGTGGTGTA	Mouse
Irf 9	CCCGAGAGAGGTCGTATGGA	TGGTTCCGTGGTTGGTTAGG	Mouse
Ptpn2	GGCCAACGGATGACAGAGAA	GGTCAGGGGTCAAACAACCA	Mouse
Gapdh	AAGGTCATCCCAGAGCTGAA	CTGCTTCACCACCTTCTTGA	Mouse

Analytical spectra for Ptpn2 inhibitors

1

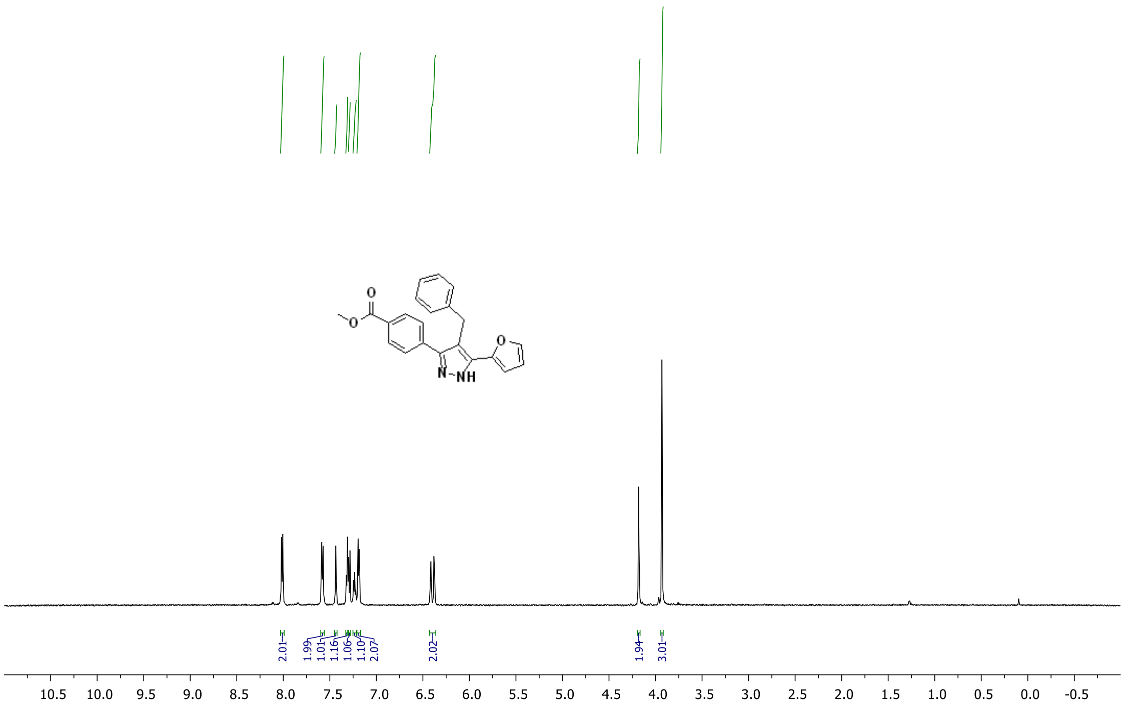

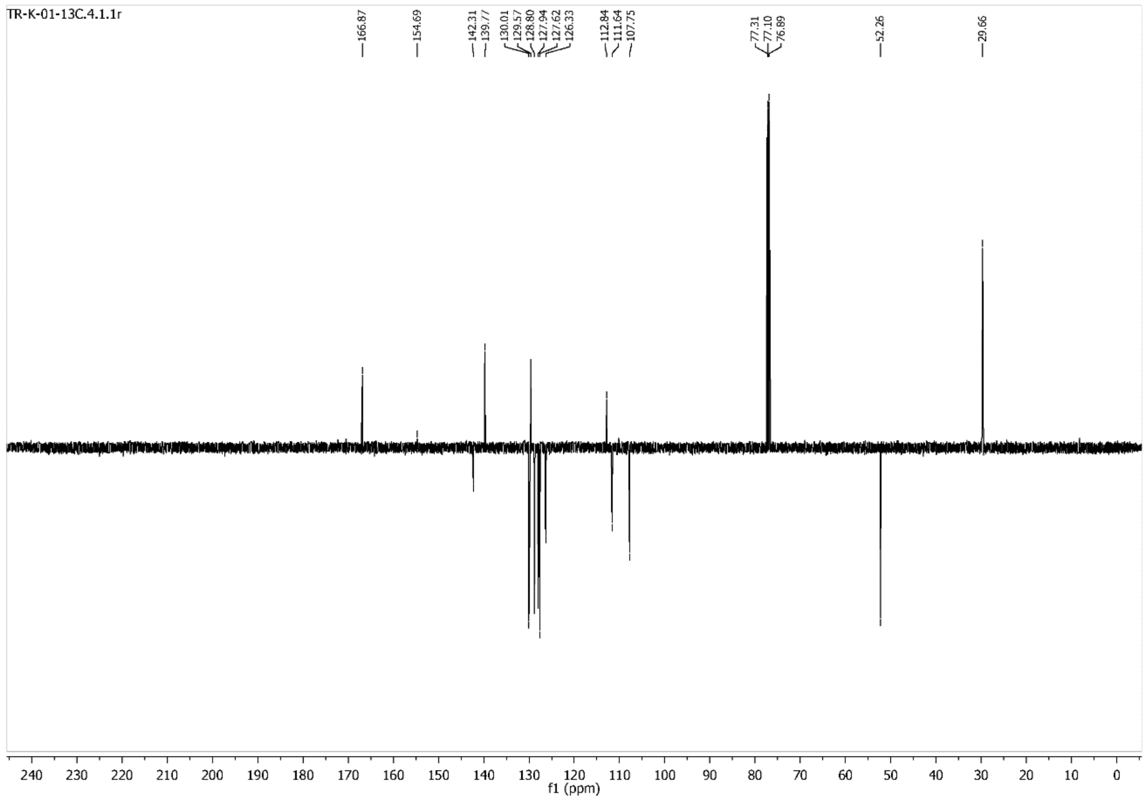
TR-K-01-13C.4.1.1r
166.87
154.69
142.31
139.77
130.01
129.57
128.80
127.94
127.62
126.33
112.84
111.64
107.75
77.31
77.10
76.89
52.26
29.66
f1 (ppm)

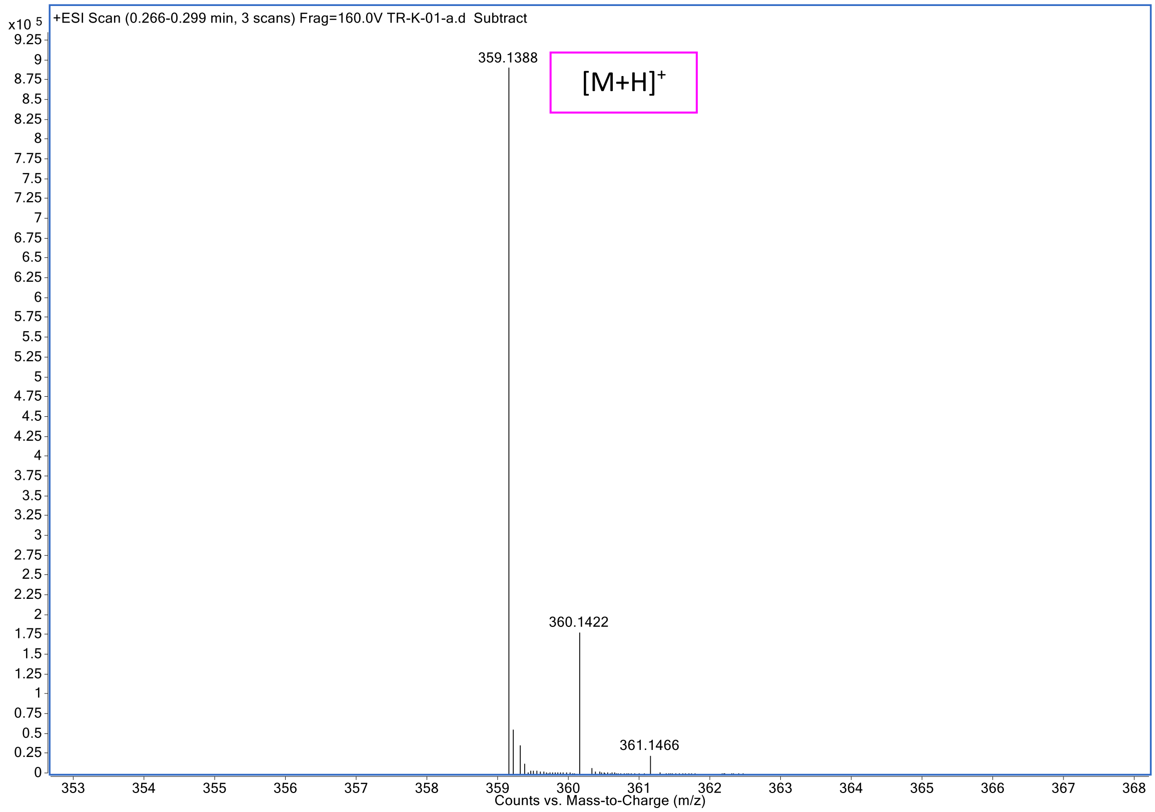
+ESI Scan (0.266-0.299 min, 3 scans) Frag=160.0V TR-K-01-a.d Subtract
x10 5
359.1388
[M+H]+
360.1422
361.1466
Counts vs. Mass-to-Charge (m/z)

2

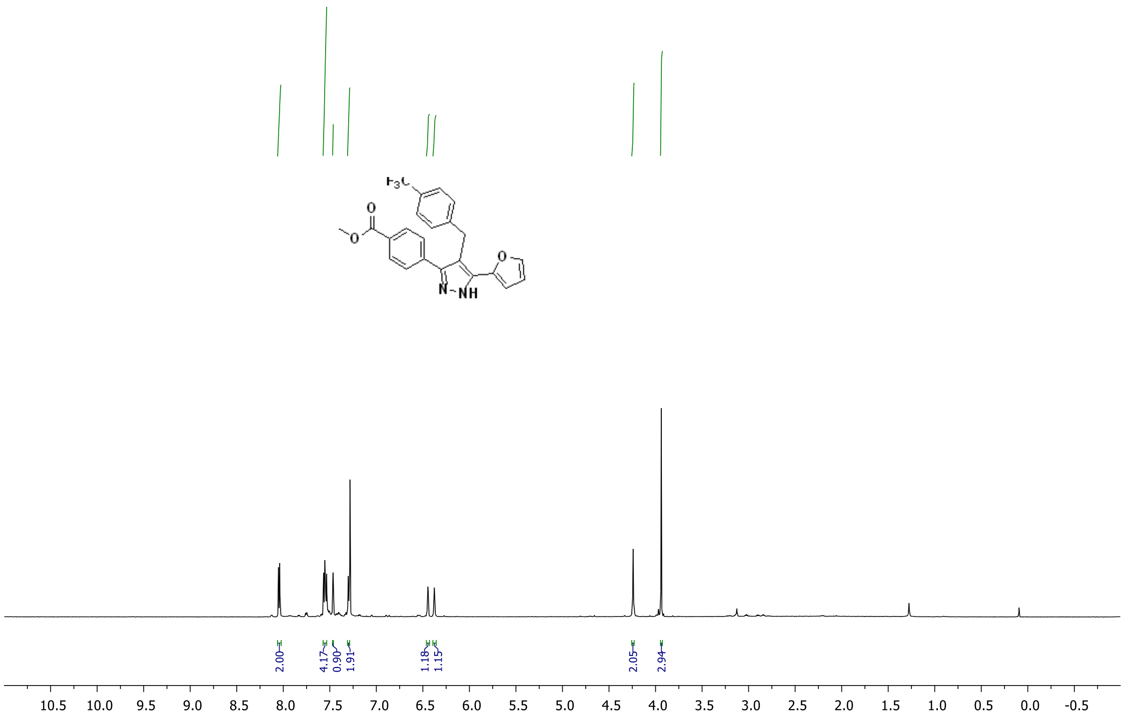

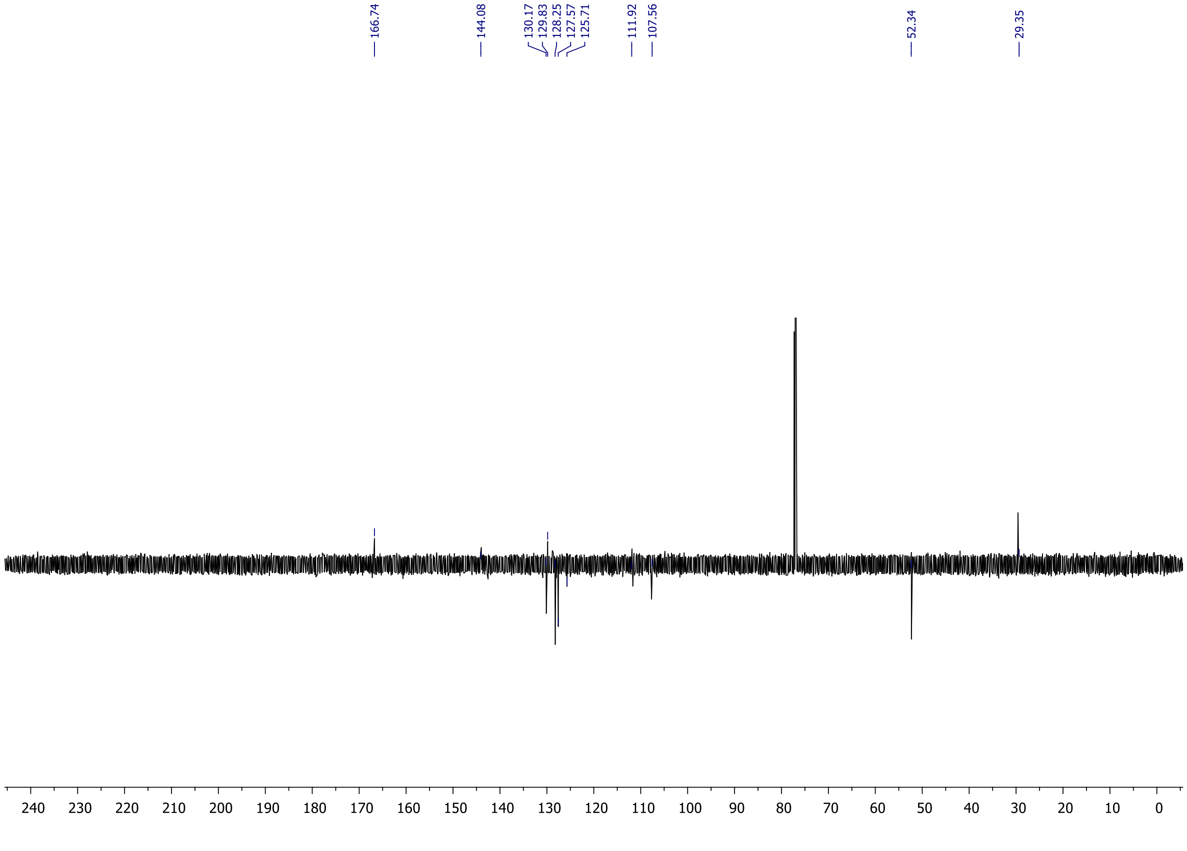

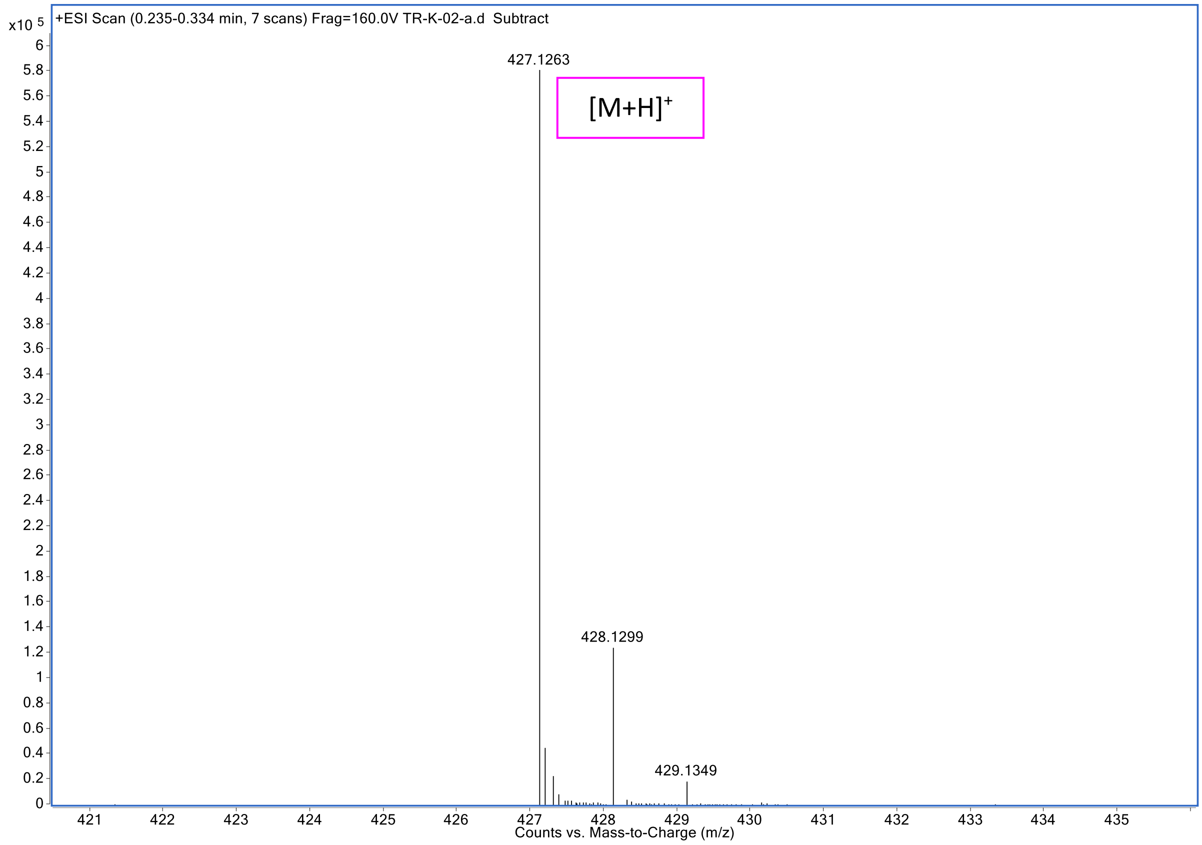
+ESI Scan (0.235-0.334 min, 7 scans) Frag=160.0V TR-K-02-a.d Subtract
x10 5
427.1263
[M+H]+
428.1299
429.1349
Counts vs. Mass-to-Charge (m/z)

3

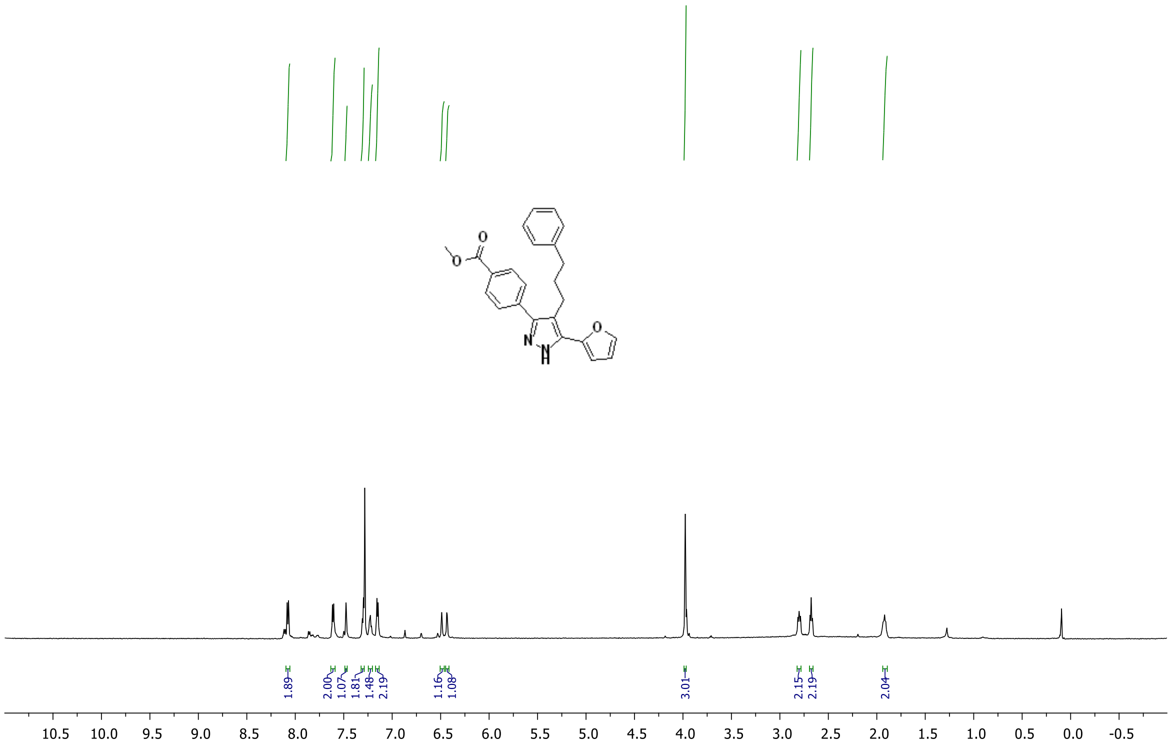
1.89
2.00
1.07
1.81
1.48
2.19
1.16
1.08
3.01
2.15
2.19
2.04
10.5 10.0 9.5 9.0 8.5 8.0 7.5 7.0 6.5 6.0 5.5 5.0 4.5 4.0 3.5 3.0 2.5 2.0 1.5 1.0 0.5 0.0 -0.5

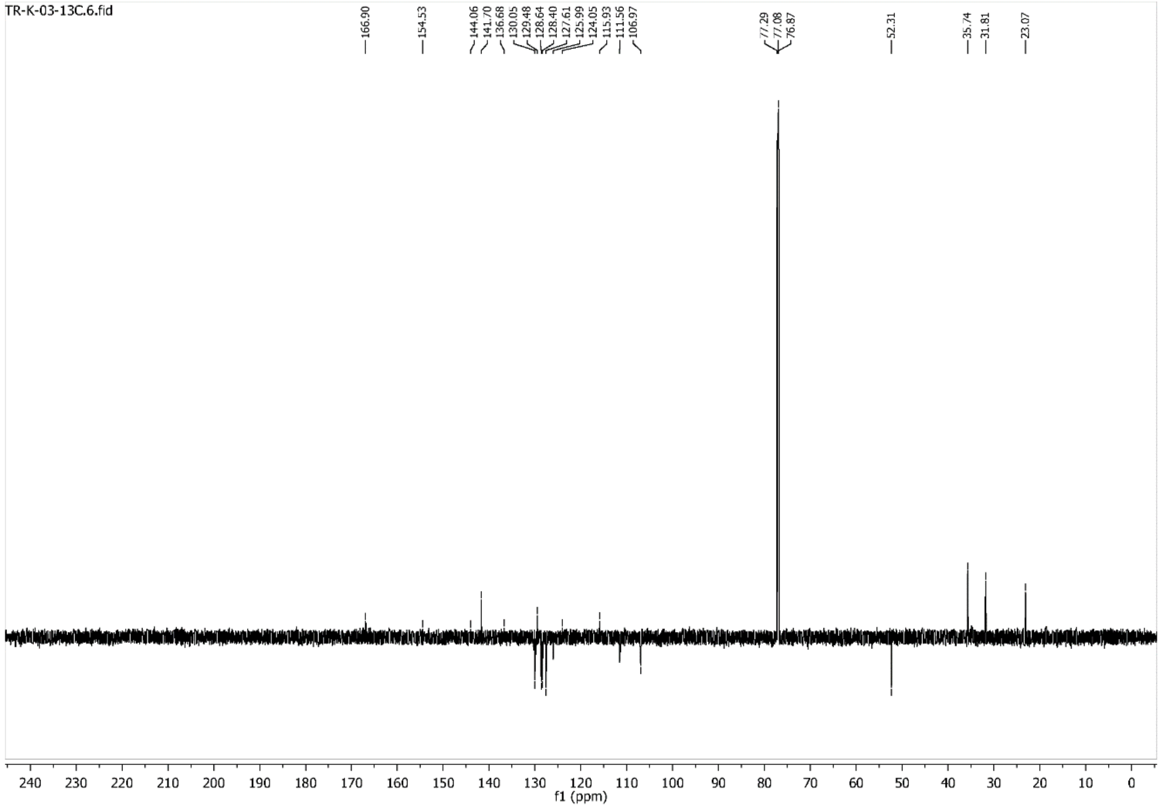
TR-K-03-13C.6.fid
166.90
154.53
144.06
141.70
136.68
130.05
129.48
128.64
128.40
127.61
125.99
124.05
115.93
111.56
106.97
77.29
77.08
76.87
52.31
35.74
31.81
23.07
240 230 220 210 200 190 180 170 160 150 140 130 120 110 100 90 80 70 60 50 40 30 20 10 0
f1 (ppm)

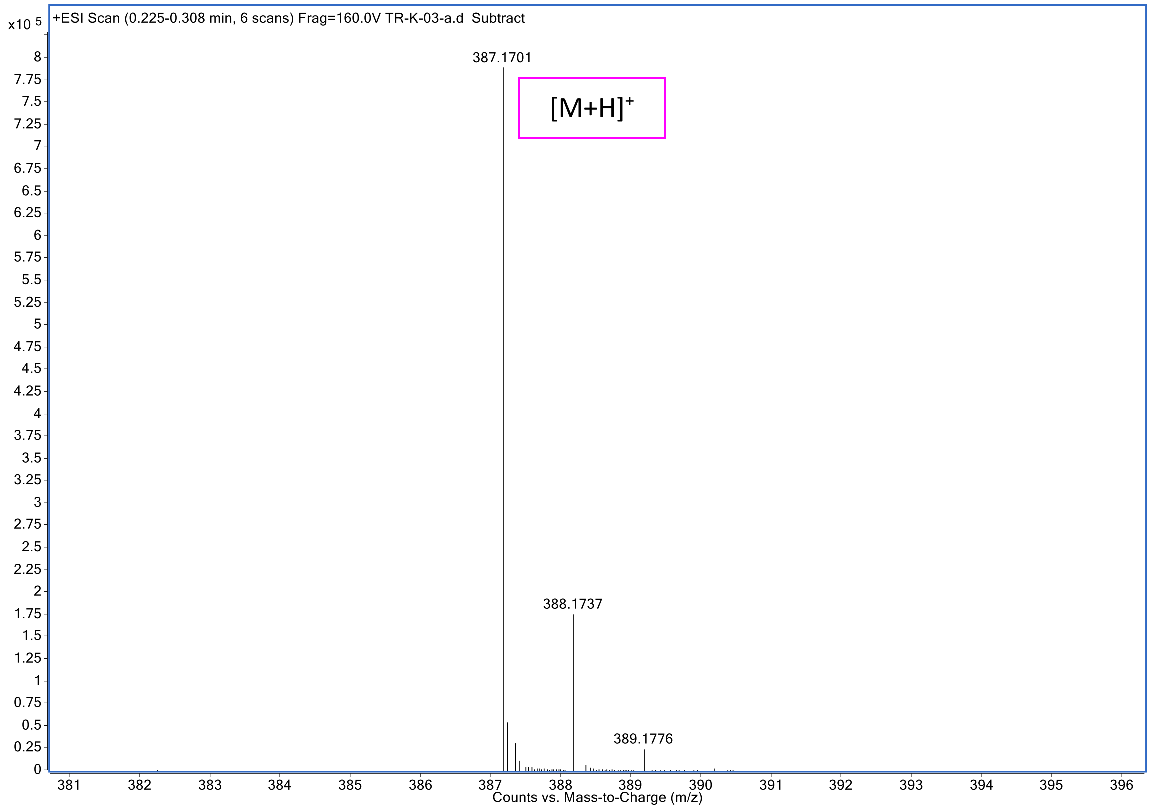
+ESI Scan (0.225-0.308 min, 6 scans) Frag=160.0V TR-K-03-a.d Subtract
387.1701
[M+H]+
388.1737
389.1776
Counts vs. Mass-to-Charge (m/z)

4

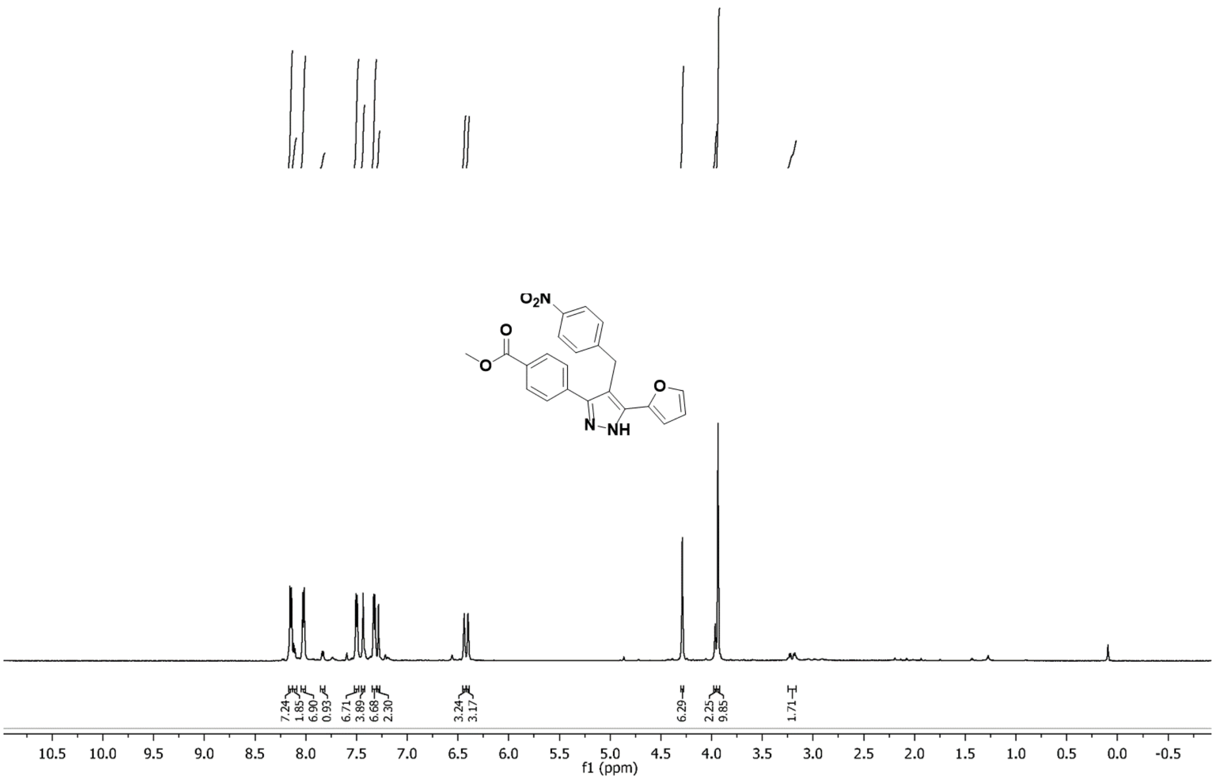
O2N
O
O
N
NH
O
f1 (ppm)

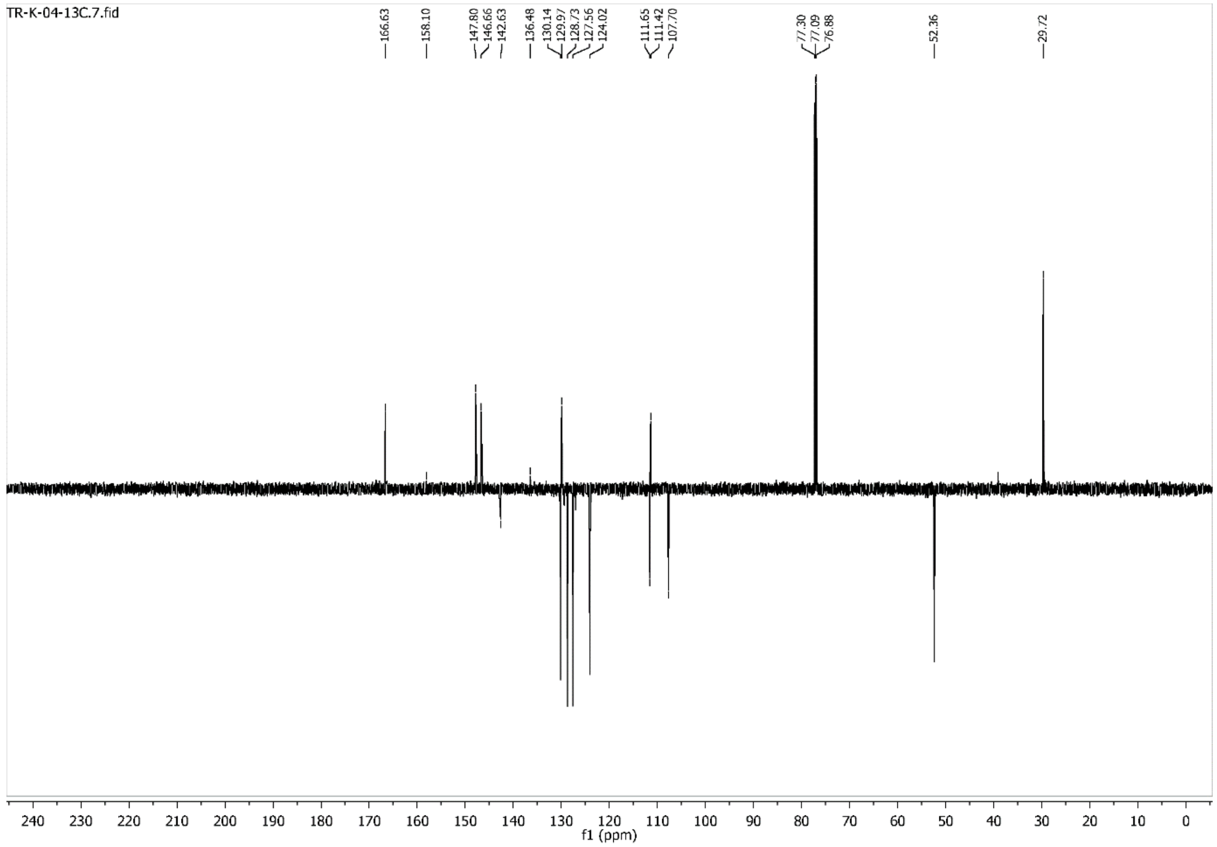
TR-K-04-13C.7.fid
166.63
158.10
147.80
146.66
142.63
136.48
130.14
129.97
128.73
127.56
124.02
111.65
111.42
107.70
77.30
77.09
76.88
52.36
29.72
f1 (ppm)

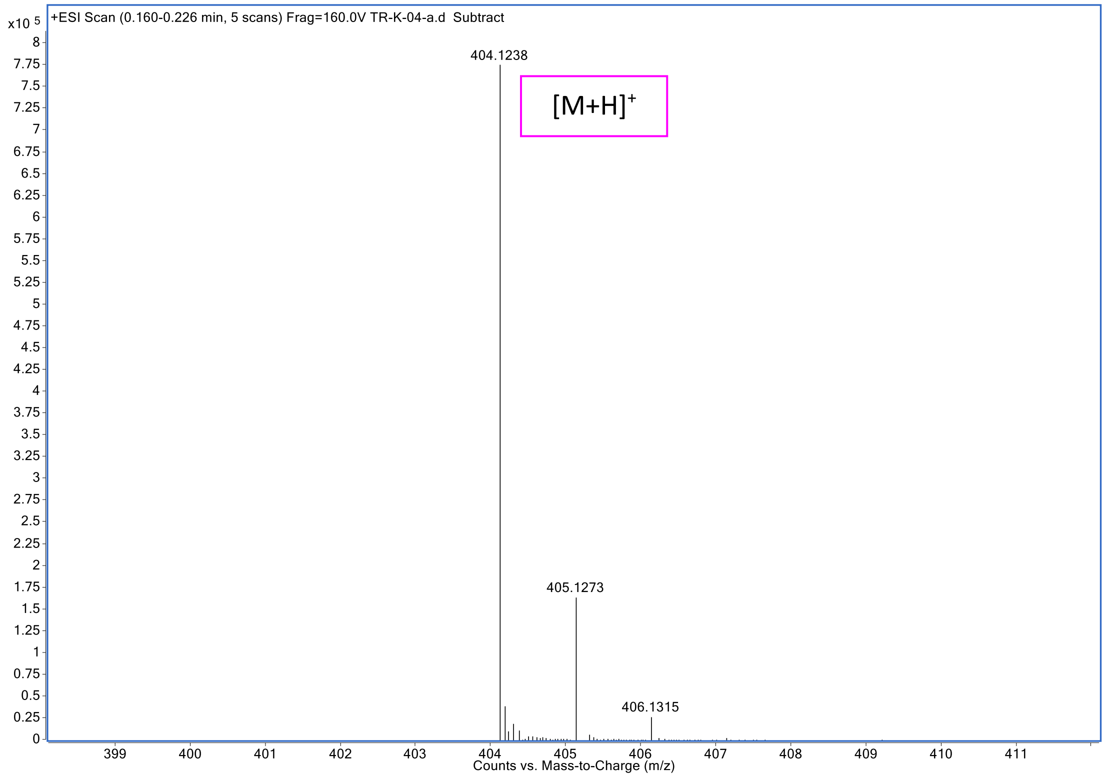
+ESI Scan (0.160-0.226 min, 5 scans) Frag=160.0V TR-K-04-a.d Subtract
x10 5
404.1238
[M+H]+
405.1273
406.1315
Counts vs. Mass-to-Charge (m/z)

5

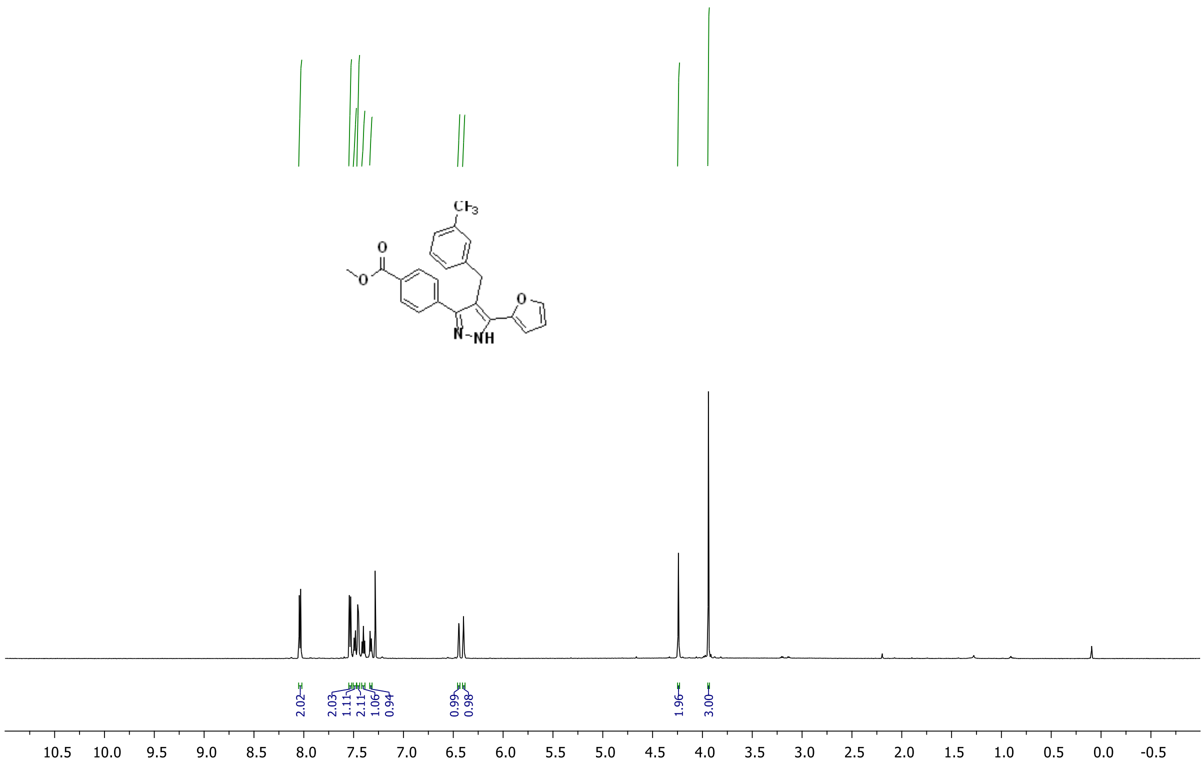

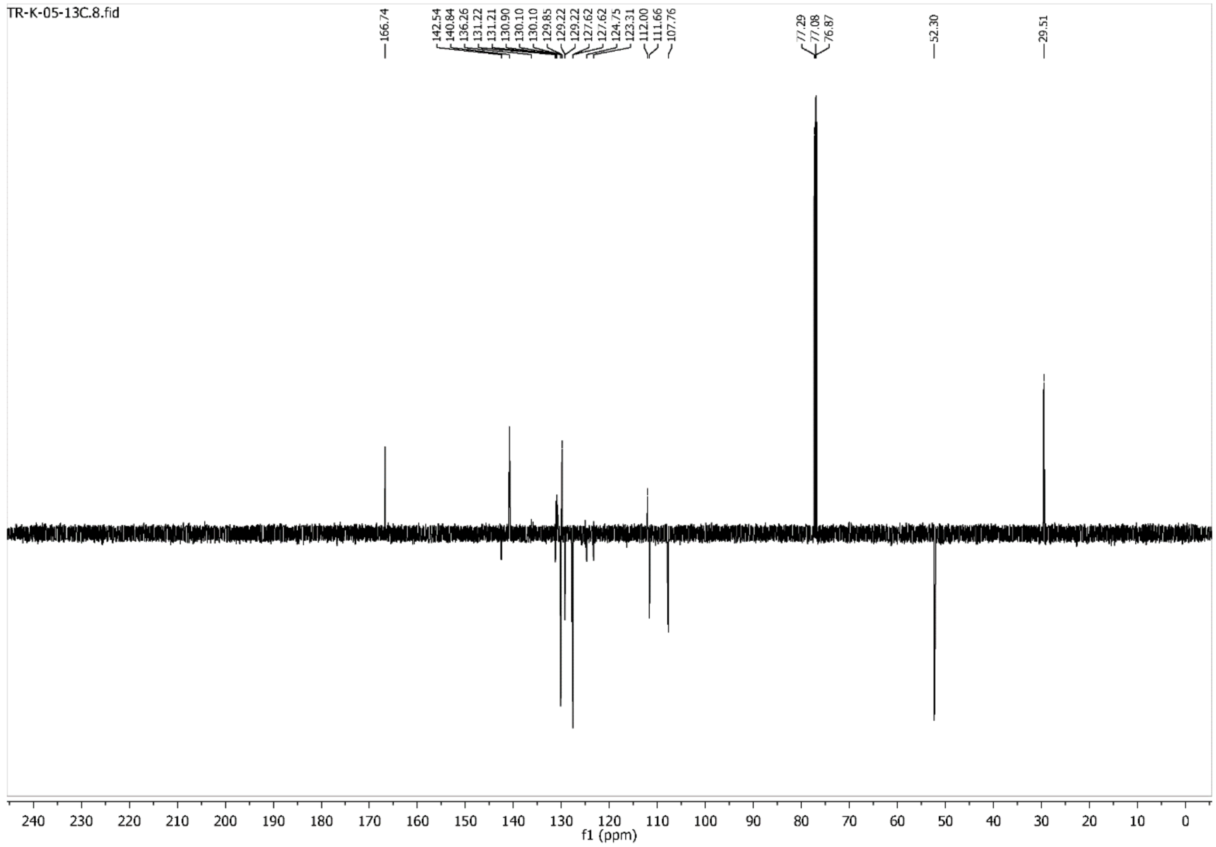
TR-K-05-13C.8.fid
166.74
142.54
140.84
136.26
131.22
131.21
130.90
130.10
130.10
129.85
129.22
129.22
129.22
127.62
127.62
124.75
123.31
112.00
111.66
107.76
77.29
77.08
76.87
52.30
29.51
f1 (ppm)

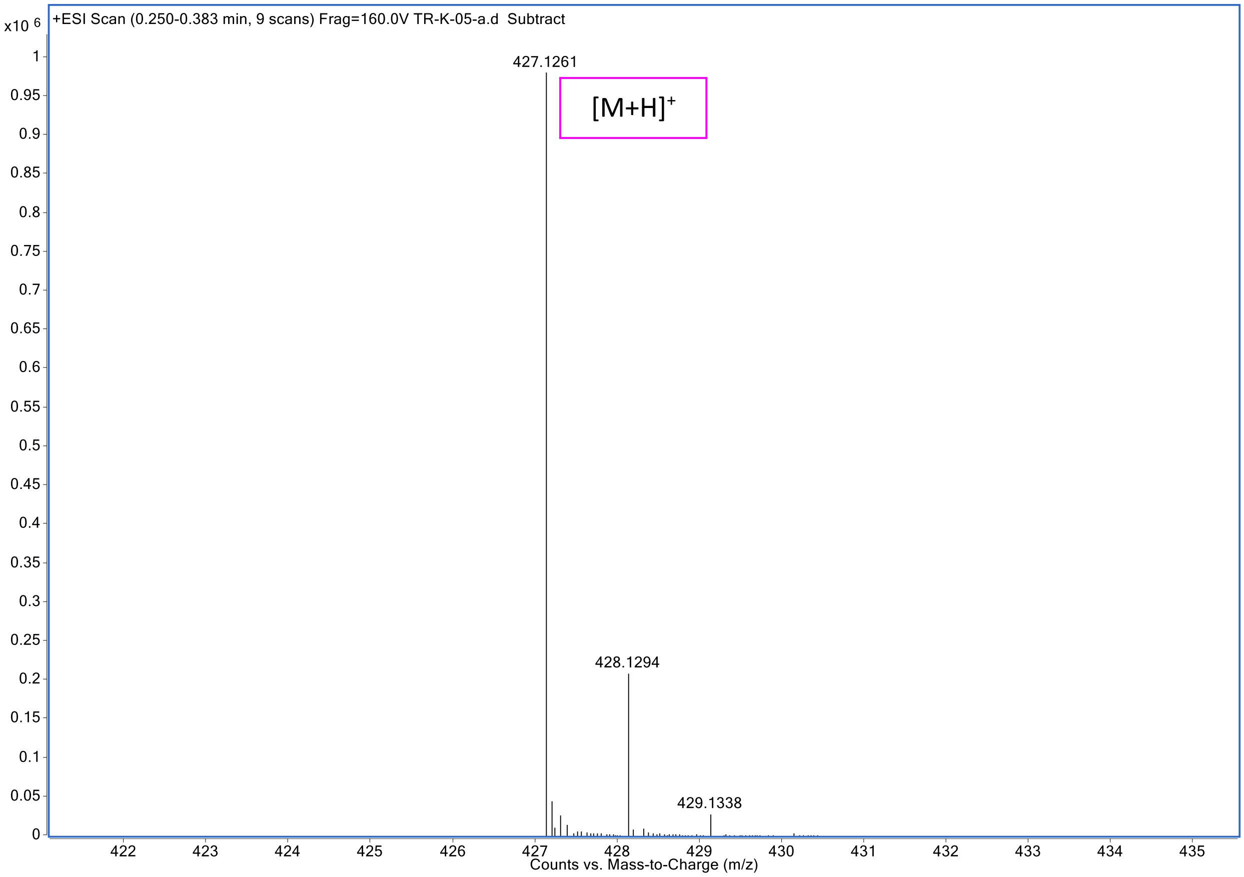
x10 6
+ESI Scan (0.250-0.383 min, 9 scans) Frag=160.0V TR-K-05-a.d Subtract
427.1261
[M+H]+
428.1294
429.1338
1
0.95
0.9
0.85
0.8
0.75
0.7
0.65
0.6
0.55
0.5
0.45
0.4
0.35
0.3
0.25
0.2
0.15
0.1
0.05
0
422
423
424
425
426
427
428
429
430
431
432
433
434
435
Counts vs. Mass-to-Charge (m/z)

6

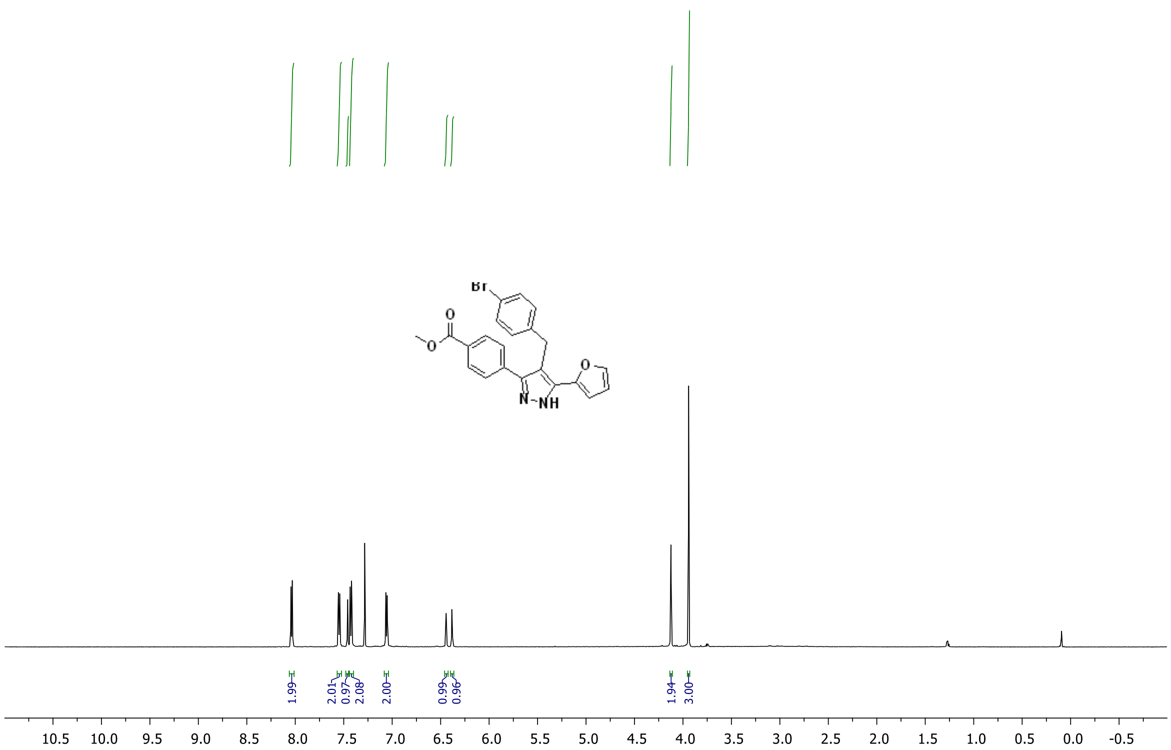

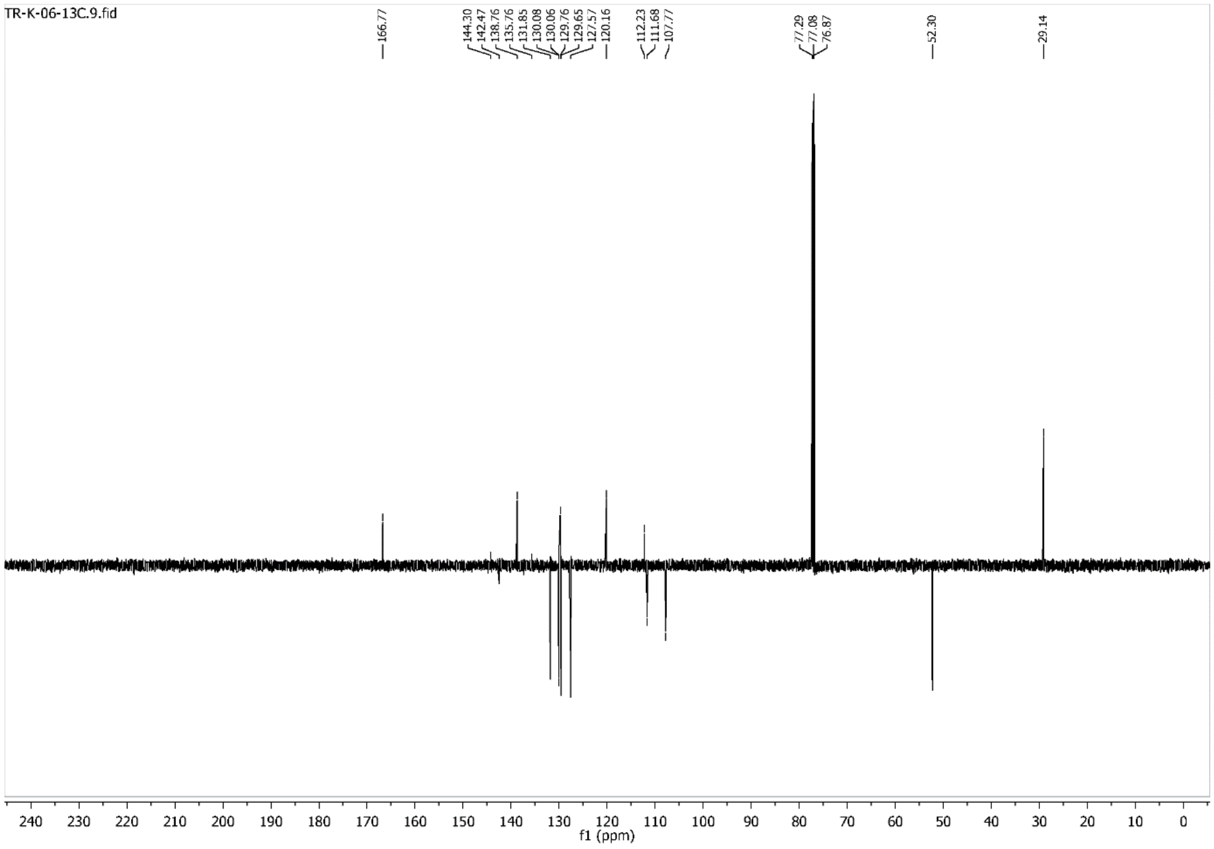
TR-K-06-13C.9.fid
f1 (ppm)

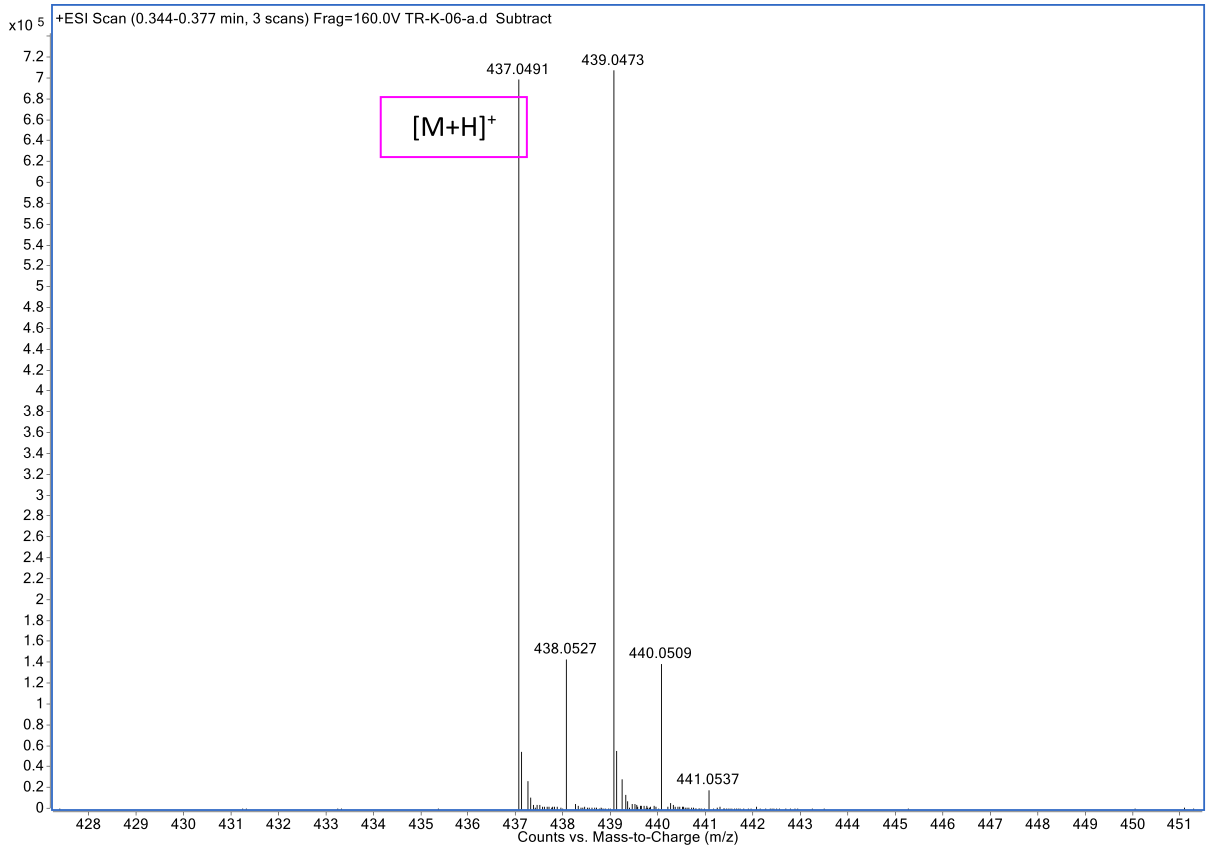

x10 5
+ESI Scan (0.344-0.377 min, 3 scans) Frag=160.0V TR-K-06-a.d Subtract
437.0491
439.0473
[M+H]+
438.0527
440.0509
441.0537
Counts vs. Mass-to-Charge (m/z)

7

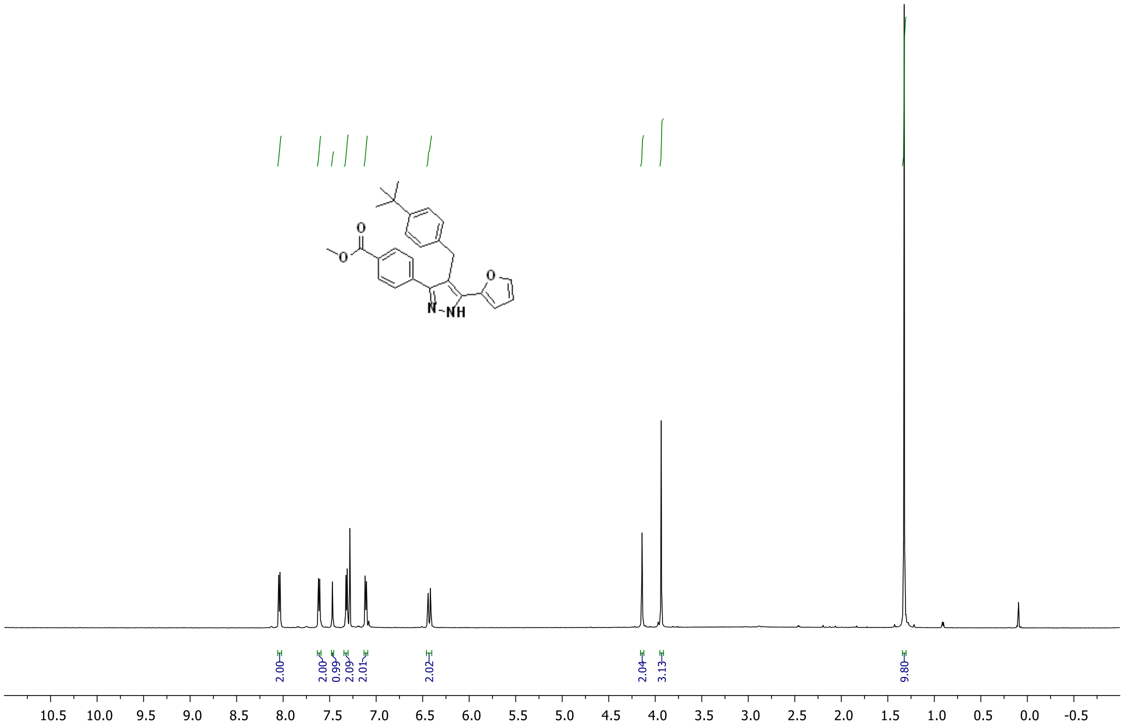

N-NH
O
2.00
2.00
0.99
2.09
2.01
2.02
2.04
3.13
9.80
10.5 10.0 9.5 9.0 8.5 8.0 7.5 7.0 6.5 6.0 5.5 5.0 4.5 4.0 3.5 3.0 2.5 2.0 1.5 1.0 0.5 0.0 -0.5

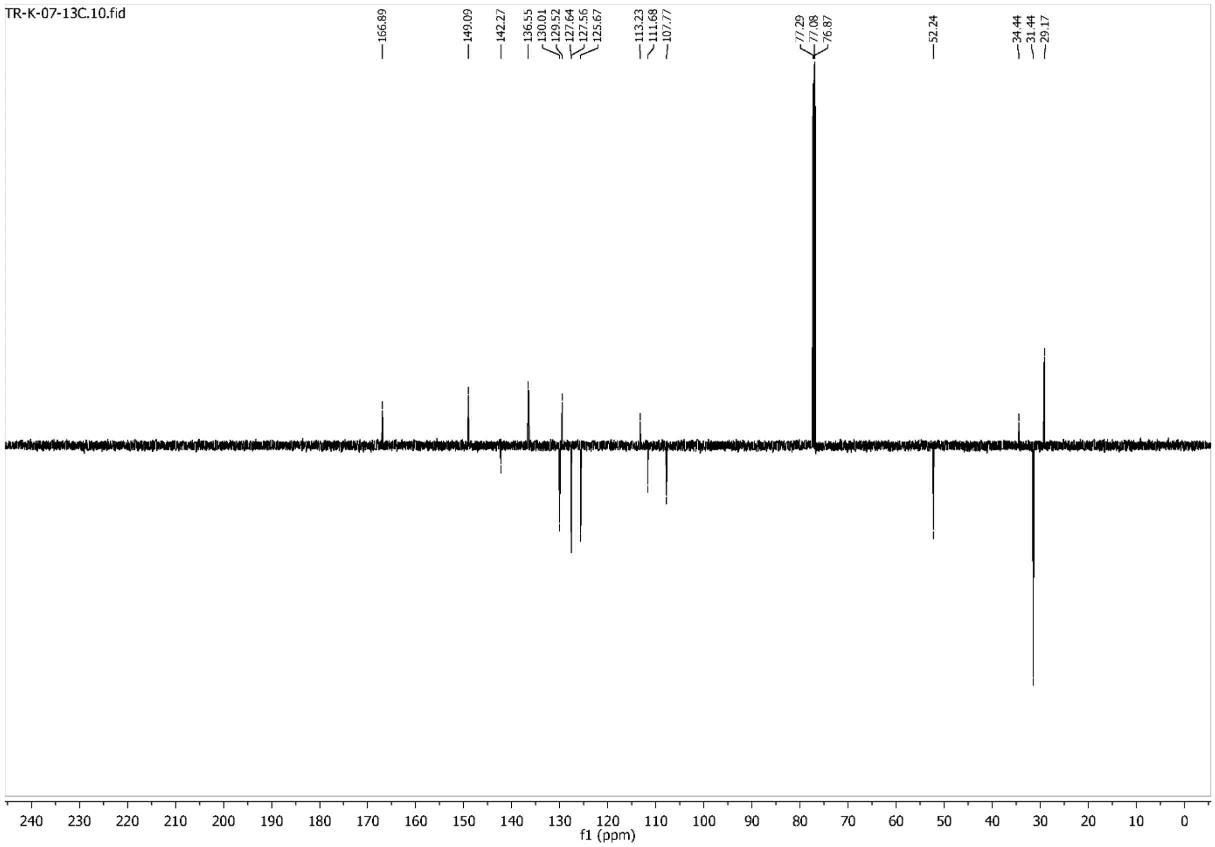

TR-K-07-13C.10.fid
166.89
149.09
142.27
136.55
130.01
129.52
127.64
127.56
125.67
113.23
111.68
107.77
77.29
77.08
76.87
52.24
34.44
31.44
29.17
240 230 220 210 200 190 180 170 160 150 140 130 120 110 100 90 80 70 60 50 40 30 20 10 0
f1 (ppm)

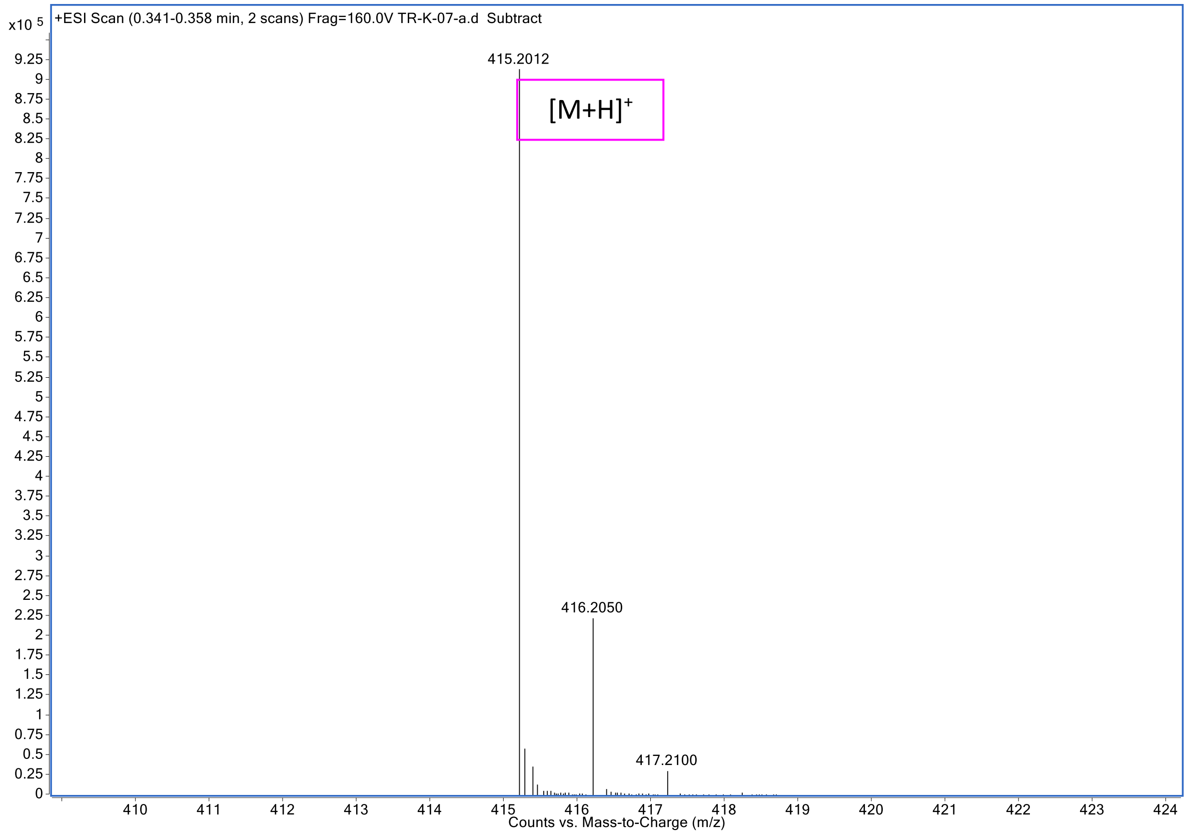

+ESI Scan (0.341-0.358 min, 2 scans) Frag=160.0V TR-K-07-a.d Subtract
x10 5
415.2012
[M+H]+
416.2050
417.2100
Counts vs. Mass-to-Charge (m/z)

8

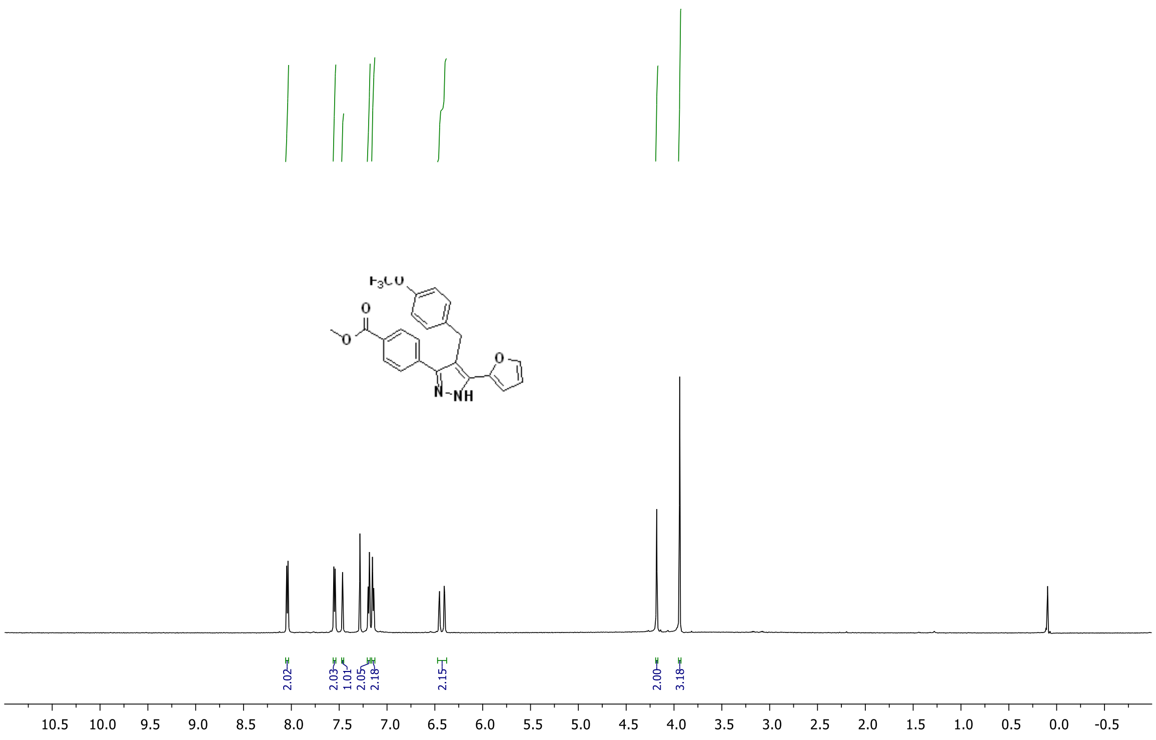

F_3CO
O
O
N
NH
O
2.02
2.03
1.01
2.05
2.18
2.15
2.00
3.18
10.5 10.0 9.5 9.0 8.5 8.0 7.5 7.0 6.5 6.0 5.5 5.0 4.5 4.0 3.5 3.0 2.5 2.0 1.5 1.0 0.5 0.0 -0.5

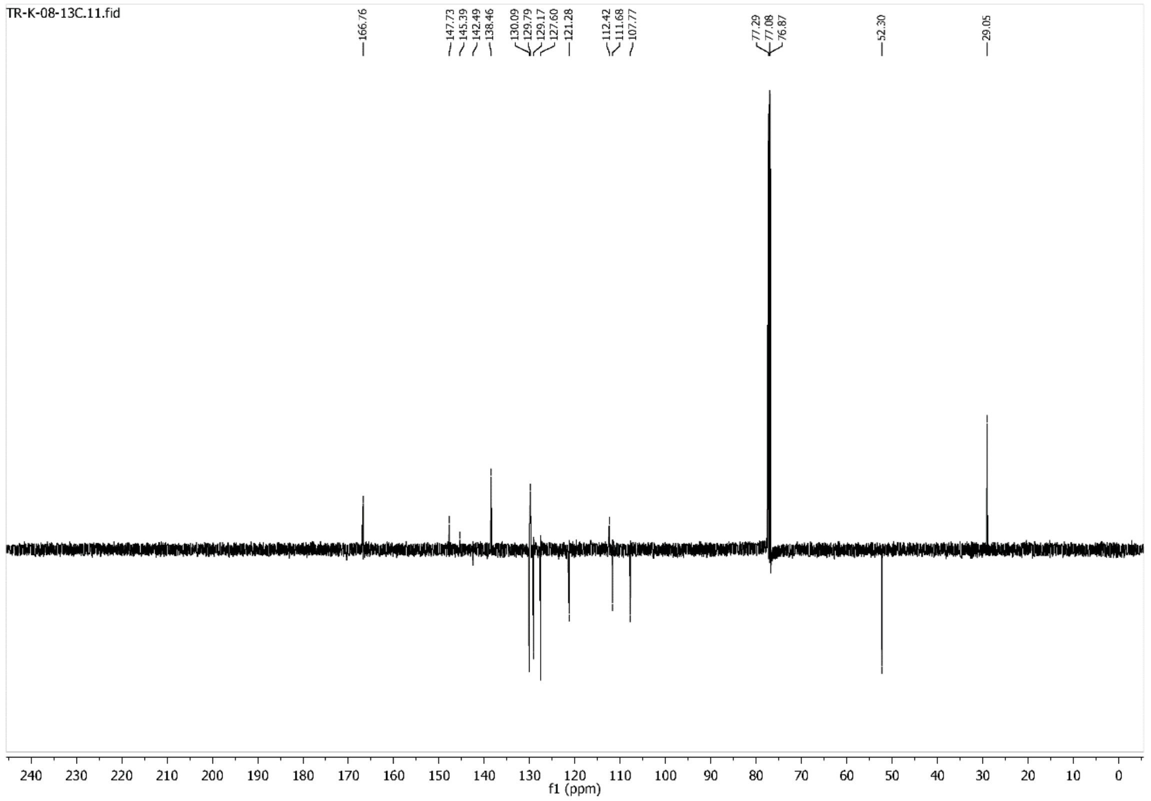

TR-K-08-13C.11.fid
166.76
147.73
145.39
142.49
138.46
130.09
129.79
129.17
127.60
121.28
112.42
111.68
107.77
77.29
77.08
76.87
52.30
29.05
240 230 220 210 200 190 180 170 160 150 140 130 120 110 100 90 80 70 60 50 40 30 20 10 0
f1 (ppm)

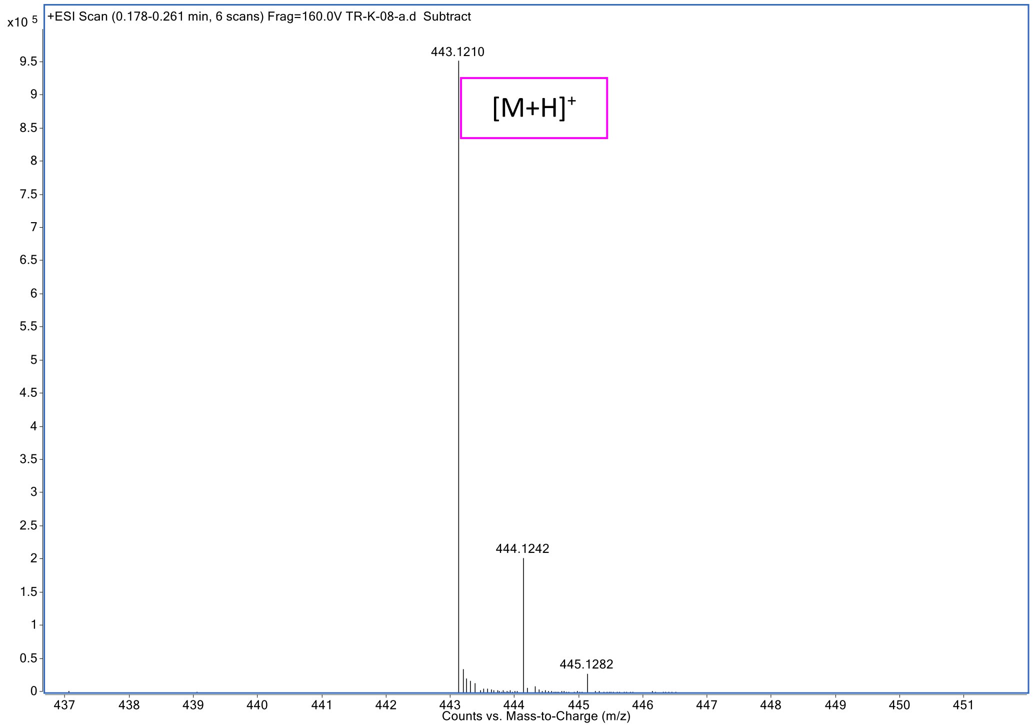

x10 5
+ESI Scan (0.178-0.261 min, 6 scans) Frag=160.0V TR-K-08-a.d Subtract
443.1210
[M+H]+
444.1242
445.1282
Counts vs. Mass-to-Charge (m/z)

9

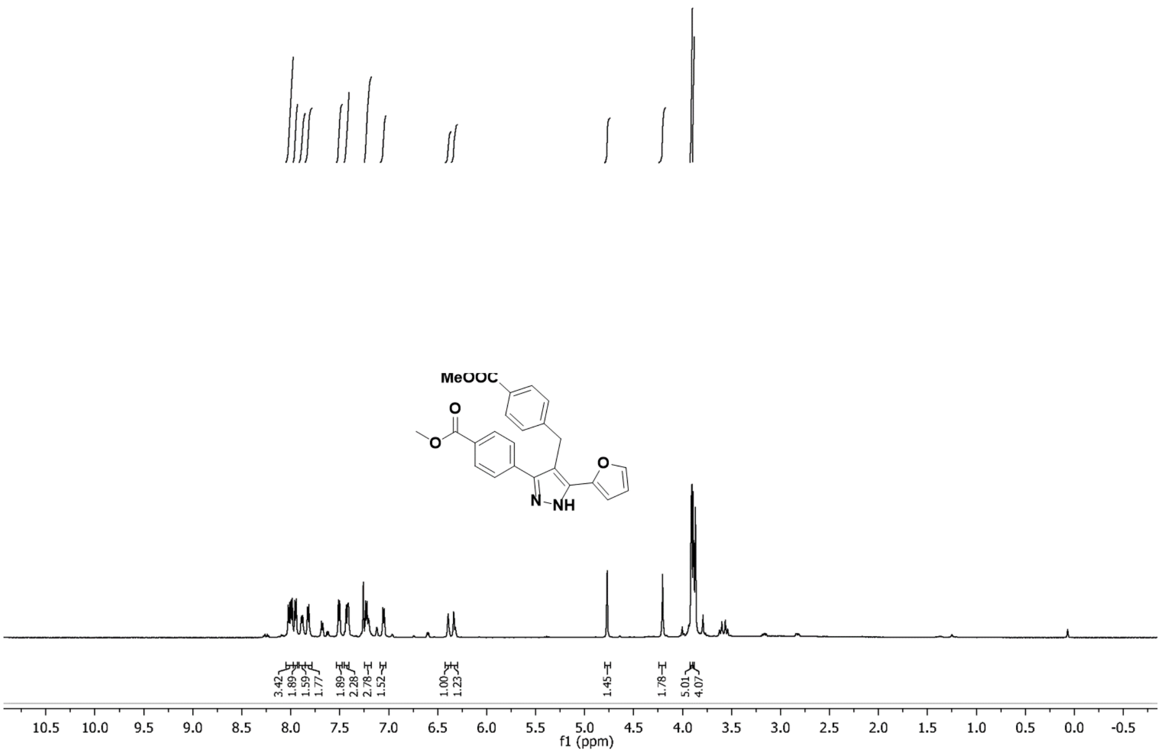

MeOOC
O
O
O
N
NH
3.42
1.89
1.59
1.77
1.89
2.28
2.78
1.52
1.00
1.23
1.45
1.78
5.01
4.07
f1 (ppm)

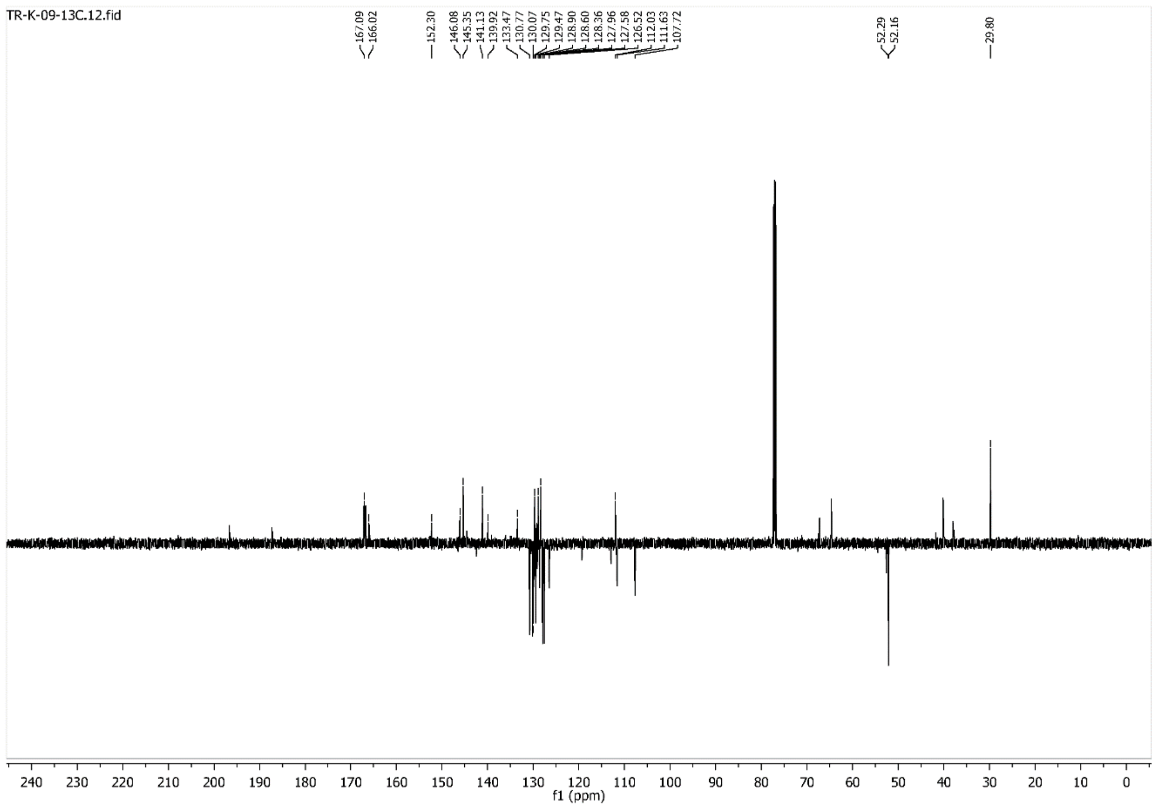
TR-K-09-13C.12.fid
167.09
166.02
152.30
146.08
145.35
141.13
139.92
133.47
130.77
130.07
129.75
129.47
128.90
128.60
128.36
127.96
127.58
126.52
112.03
111.63
107.72
52.29
52.16
29.80
240 230 220 210 200 190 180 170 160 150 140 130 120 110 100 90 80 70 60 50 40 30 20 10 0
f1 (ppm)

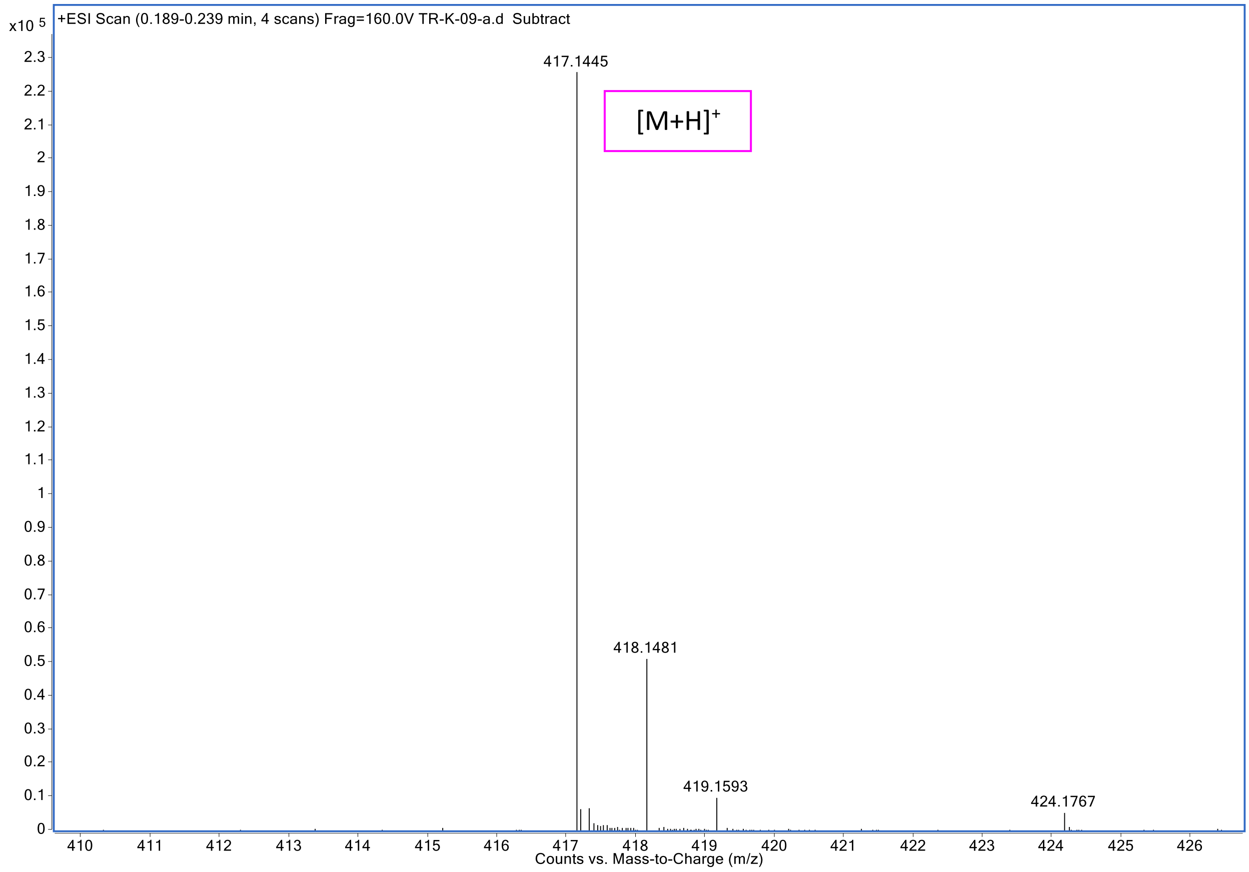
+ESI Scan (0.189-0.239 min, 4 scans) Frag=160.0V TR-K-09-a.d Subtract
x10 5
417.1445
[M+H]+
418.1481
419.1593
424.1767
Counts vs. Mass-to-Charge (m/z)

10

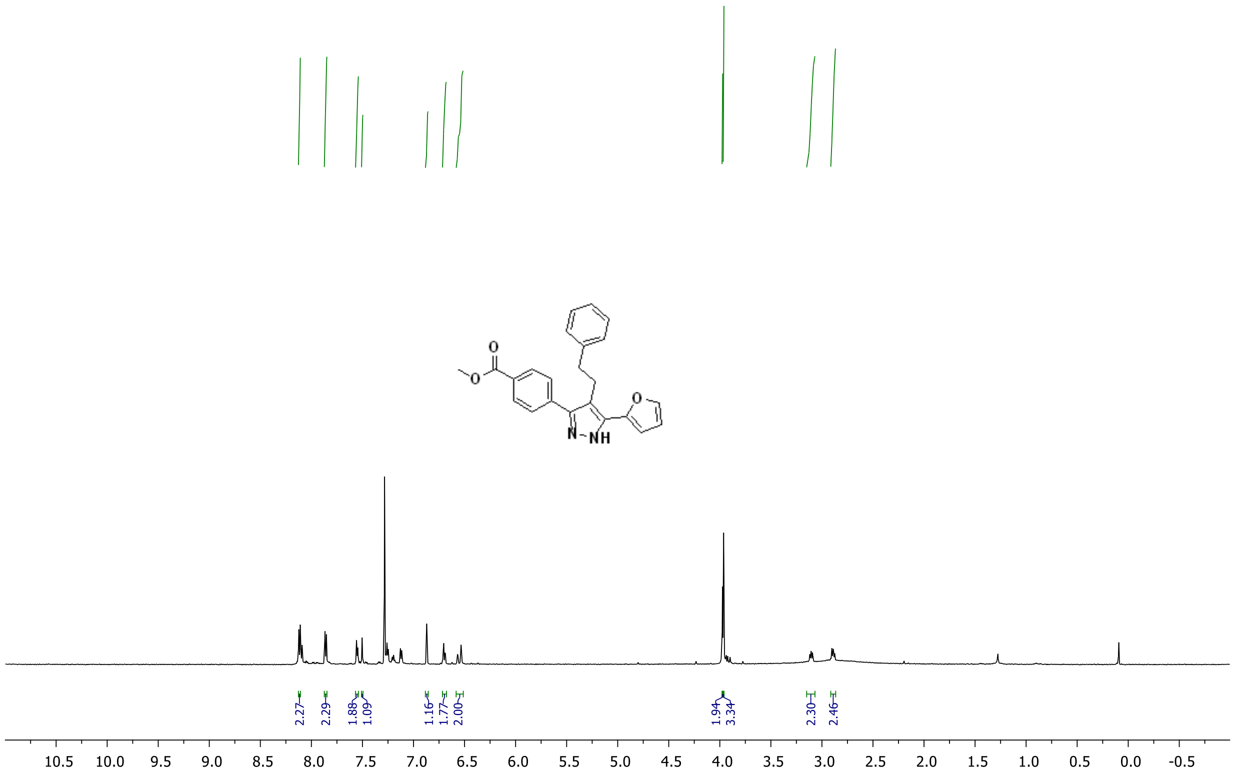

O
O
N
NH
O
2.27
2.29
1.88
1.09
1.16
1.77
2.00
1.94
3.34
2.30
2.46
10.5 10.0 9.5 9.0 8.5 8.0 7.5 7.0 6.5 6.0 5.5 5.0 4.5 4.0 3.5 3.0 2.5 2.0 1.5 1.0 0.5 0.0 -0.5

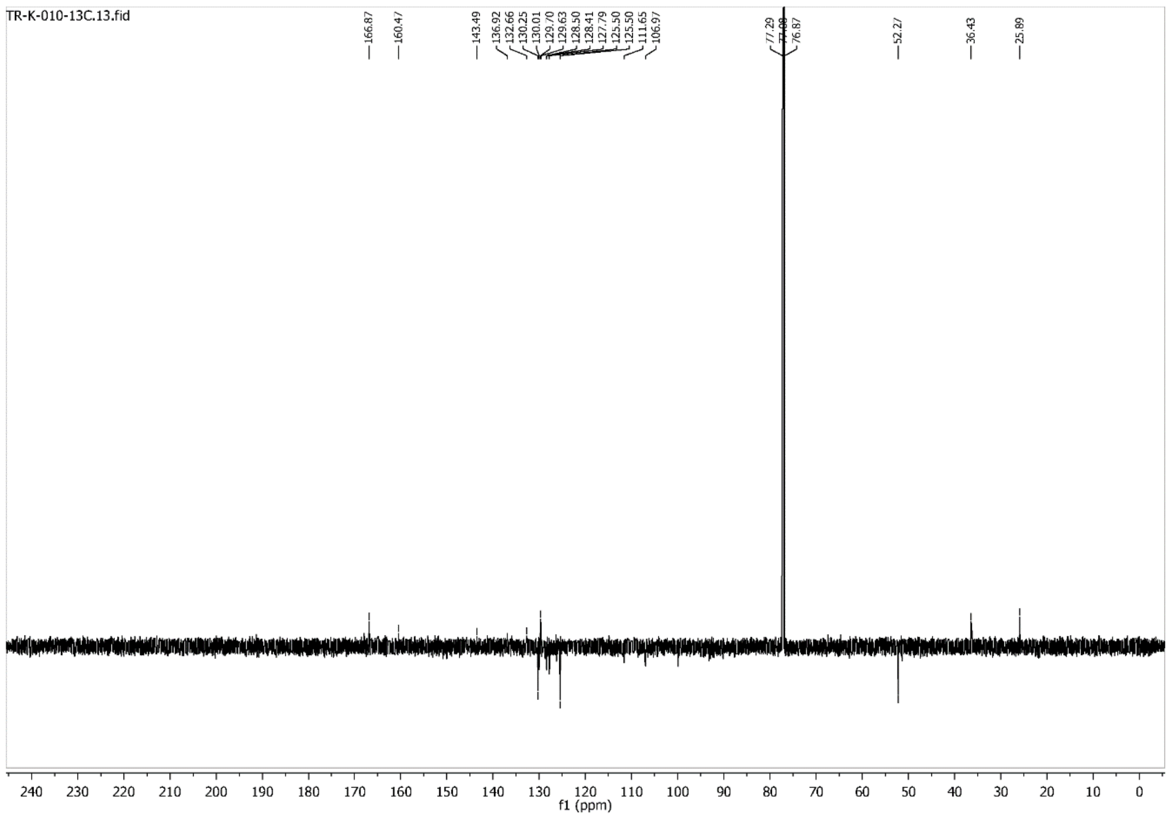

TR-K-010-13C.13.fid
166.87
160.47
143.49
136.92
132.66
130.25
130.01
129.70
129.63
128.50
128.41
127.79
125.50
125.50
111.65
106.97
77.29
76.87
52.27
36.43
25.89
f1 (ppm)

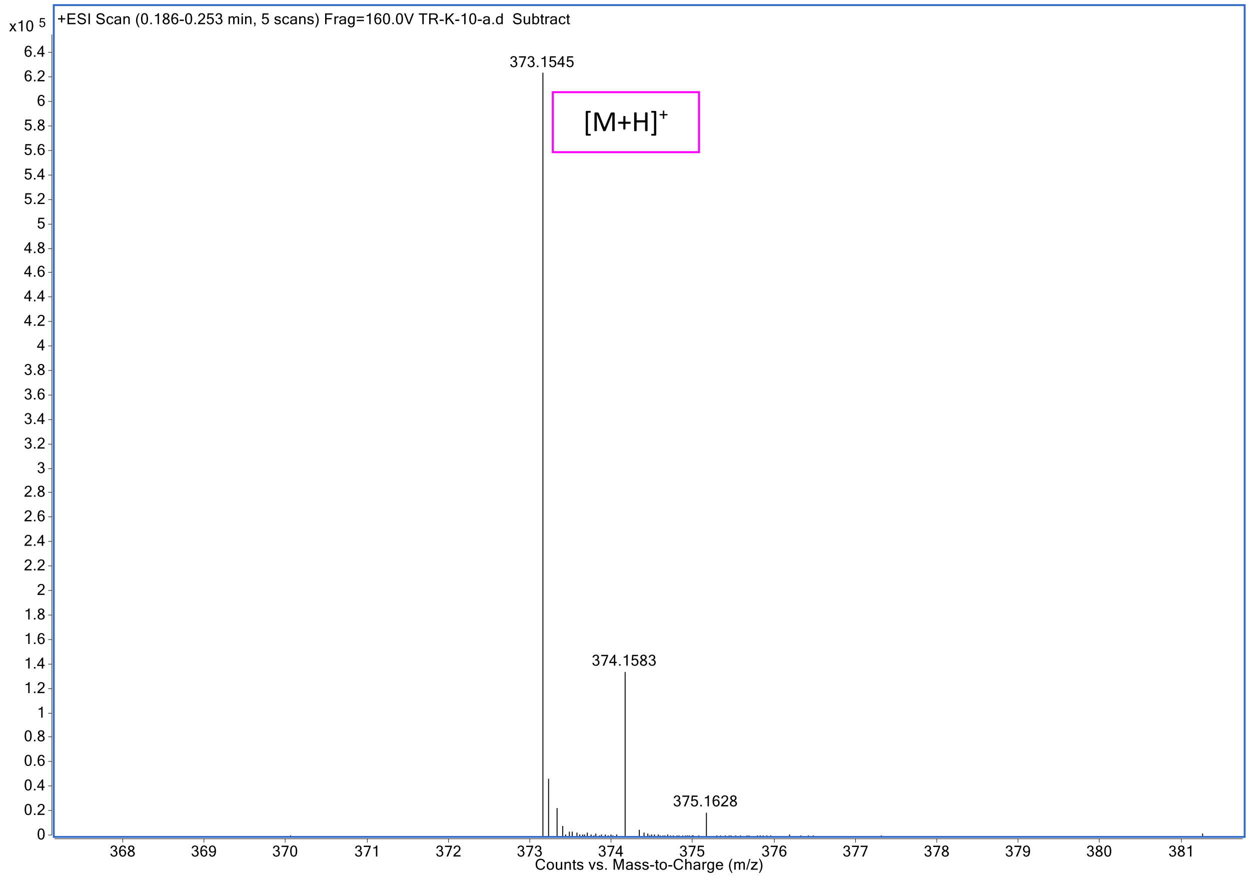

+ESI Scan (0.186-0.253 min, 5 scans) Frag=160.0V TR-K-10-a.d Subtract
x10 5
373.1545
[M+H]+
374.1583
375.1628
Counts vs. Mass-to-Charge (m/z)

References

1 Iversen, L. F. *et al.* Structure determination of T cell protein-tyrosine phosphatase. *J Biol Chem* **277**, 19982-19990, doi:10.1074/jbc.M200567200 (2002).

2 Jacobson, M. P., Friesner, R. A., Xiang, Z. & Honig, B. On the role of the crystal environment in determining protein side-chain conformations. *J Mol Biol* **320**, 597-608, doi:10.1016/s0022-2836(02)00470-9 (2002).

3 Jacobson, M. P. *et al.* A hierarchical approach to all-atom protein loop prediction. *Proteins* **55**, 351-367, doi:10.1002/prot.10613 (2004).

4 Friesner, R. A. *et al.* Glide: a new approach for rapid, accurate docking and scoring. 1. Method and assessment of docking accuracy. *J Med Chem* **47**, 1739-1749, doi:10.1021/jm0306430 (2004).

5 Halgren, T. A. *et al.* Glide: a new approach for rapid, accurate docking and scoring. 2. Enrichment factors in database screening. *J Med Chem* **47**, 1750-1759, doi:10.1021/jm030644s (2004).

6 Friesner, R. A. *et al.* Extra precision glide: docking and scoring incorporating a model of hydrophobic enclosure for protein-ligand complexes. *J Med Chem* **49**, 6177-6196, doi:10.1021/jm051256o (2006).
